# A chemosensor-based chiral coassembly with switchable circularly polarized luminescence

**DOI:** 10.1038/s41467-021-26700-2

**Published:** 2021-11-03

**Authors:** Qiuhong Cheng, Aiyou Hao, Pengyao Xing

**Affiliations:** Key Laboratory of Colloid and Interface Chemistry of Ministry of Education and School of Chemistry and Chemical Engineering, Shandong University, Jinan, 250100 People’s Republic of China

**Keywords:** Organic molecules in materials science, Self-assembly, Self-assembly

## Abstract

Fluorescent chemosensors represent fast response to analytes with pronounced luminescent variations. They are promising as potential candidates in controlling luminescence and chiroptical activities of self-assembled chiral systems, which however have not been accomplished to date. We present a coassembled multiple component system that could respond to SO_2_ derivatives, giving rise to dynamic aggregation behaviors and switchable luminescence as well as circularly polarized luminescence (CPL). Cholesteryl-naphthalimide and coumarin derivatives coassemble into vesicles and nanohelices under the solvent strategy, behaving as energy transfer donor and accepter respectively. Energy transfer enables CPL transition from green to red depending on the molar fraction. After the addition of SO_2_ derivatives, hypochromic shifts occur to CPL due to the nucleophilic addition reaction to coumarin domain, hindering energy transfer and allow for the emergence of pristine luminescence. Here, we show a protocol to control over luminescence and chiroptical features of supramolecular chiral self-assemblies using fluorescent chemosensors.

## Abstract (web summary)

‘Fluorescent chemisensors with fast response time and pronounced luminescence variation are important but have not been well investigated for self-assembled chiral systems. Here, the authors present a coassembled multiple component system that responds to SO_2_ derivatives, giving rise to dynamic aggregation behaviors and switchable luminescence as well as circularly polarized luminescence.

## Introduction

As one of the most intriguing properties of chiral species, chiroptical activity represents polarized optical features, which normally include circular dichroism (CD), circularly polarized luminescence (CPL), vibrational circular dichroism (VCD), and Raman optical activity (ROA)^1–6^. These spectroscopy techniques are widely applied to the determination of absolute configuration, chiral sensing, detection, display, and information applications^7–11^. Among them, CPL is emerging recently due to it is key in the next generation of three-dimensional display materials^10–14^. CPL, of which counterpart is luminescence, stands for the differential of left- and right-handed circularly polarized light emitted from chiral entities^13,15^. Over the last decades, intrinsic chiral luminescent materials covering coordination clusters^16^, perovskites^17^, helical polymers^18^, organic compounds^19^, and quantum dots^20^ have been developed. Apart from the intrinsic chiral species, self-assembly of single or multiple component coassemblies with luminescent materials is also a promising protocol in the effective fabrication of CPL active systems^21–24^. Development of CPL properties and behaviors such as upconverting, two-photon, phosphorescence, and rational control CPL wavelength, handedness, boosting the dissymmetry g-factor remain key challenges^25–28^. Utilizing stimulus-responsiveness of dynamic supramolecular self-assembly could realize a smart control over CPL properties, which however have been largely unexplored^29,30^.

Compared to the normal stimulus-responsiveness, fluorescent sensing platforms are capable of rapidly responding to analytes with dramatic variations in emission intensity or colors^31,32^. Different fluorescent mechanisms including photo-induced electron transfer^33^, fluorescent resonance energy transfer^34^, and excited-state intramolecular proton transfer^35^ are accounted for the fluorescence changes^36^. The intensive development of chemosensors probes a wide scope of analytes including anions, cations, pH, small organic compounds, HClO, SO_2_, and other important biological molecules, which interact with sensors via addition, elimination, and coordination reactions to change the structures and luminescent properties^37,38^. Chemosensors expand to biosensing with excellent applications in diagnosis^39,40^. Considering the merits of rapid responding, quantitative reaction and significant variations in luminescence, the construction of stimulus-responsive chiral nanoassemblies using fluorescent chemosensors is promising in controlling luminescent properties, which however have rarely been addressed^41^. In contrast to the diluted solution phase, aggregation may influence the responding efficacy and performances, remaining considerable challenges to design and perform. Chiroptical switch refers to the reversible variation of chiroptical signals such as CD, CPL, and VCD responding to specific stimuli. Upon interacting with stimuli, chiroptical changes such as inversion, amplification, and on/off behaviors shall emerge for the molecular or supramolecular switches. Although diverse chiroptical switchable systems with fine sensitivity and reversibility have been developed, the stimulus factors are still limited to like light irradiation, pH, electric field, temperature, chemicals, and redox. In contrast, though chemosensor platforms are normally irreversible, they cover enormous analytes which involve many important small molecules in biological system. For instance, NO, NO_2_, SO_2_, H_2_S, bio-mercaptan, pH, metal ions, enzyme, anions, amino acids, bioamines, ClO^−^, explosives, biotoxins, reactive oxygen species (ROS) and many others have been detected in vitro and in vivo^31–40^. Ascribed to the scope of analytes, chemosensor platforms possess great potential in chiroptical application rather than chiroptical switches. The advantages shall facilitate the development of chiroptical chemosensors with applications in biological system with unique signaling.

**Fig. 1:**
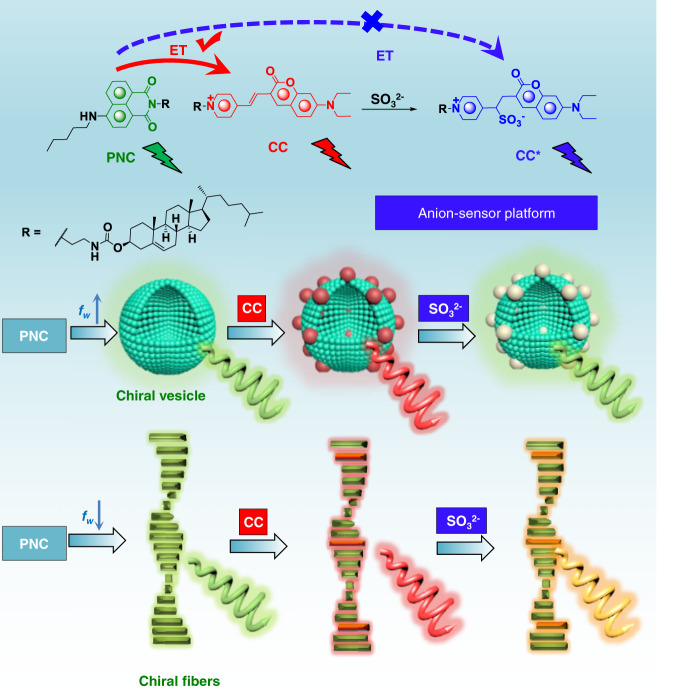
Molecular structure of PNC and CC as well as the anion-responsive energy transfer effect in vesicle and helical fiber structures mediated by water fraction (fw) (energy transfer (ET), pentylamine-substituted cholesteryl naphthalimide (PNC), cholesteryl coumarin (CC)). Blue, red, and gray particles represent PNC, CC and CC* respectively in vesicle phase. Green, red, and orange rods stand for PNC, CC and CC* respectively in chiral fiber self-assembly. Spiral arrows represent CPL with corresponding colors.

In this work, we assume that the introduction of fluorescent chemosensor into multiple component coassemblies would facilitate the fabrication of switchable luminescence and the corresponding chiroptical properties including Cotton effects and CPL. To that end, we design and synthesize pentylamine-substituted cholesteryl naphthalimide (PNC) and cholesteryl coumarin (CC) derivative which processes the same lipid domains and distinct luminophores, allowing for synergistic coassembly into liposomes and nanohelices under solvent control (Fig. 1)^42^. Naphthalimide and coumarin derivatives behaving as donor and accepter enable the energy transfer, realizing CPL evolution from green to red. In the presence of SO_2_ derivatives such as SO_3_^2−^, nucleophilic addition reaction occurs to destruct the stilbene structure, blocking the energy transfer. Consequently, recovery of CPL is accomplished. The incorporation of SO_2_ chemosensor into multiple component chiral nanoassemblies establishes an unprecedented approach to switchable luminescence and CPL in nanoassemblies. Starting from this work, more functional sensor-based chiral systems are expected under switchable and smart control manners.

## Results

### Morphology investigation

**Fig. 2:**
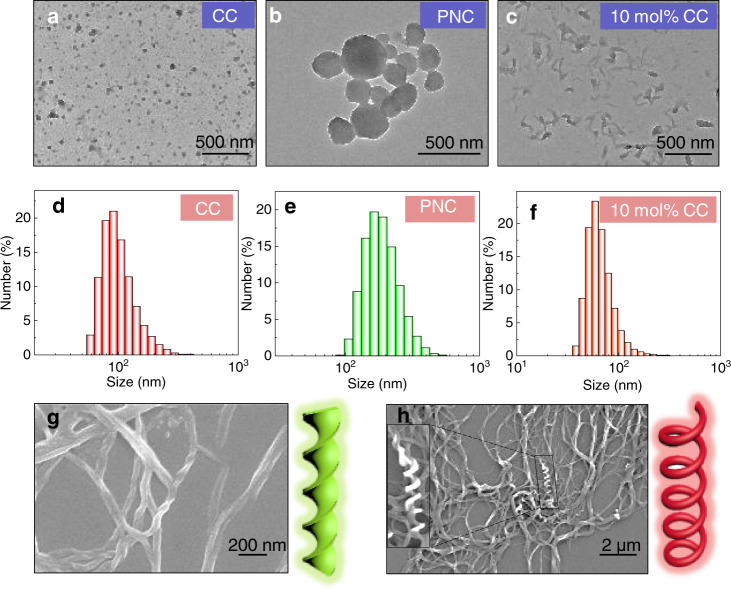
Self-assembly and coassembly of PNC and CC. TEM images of **a** CC (10^−4 ^M, THF/H_2_O = 1/9 v/v), **b** PNC (10^−4 ^M, THF/H_2_O = 1/9), **c** PNC/CC ([PNC] = 10^−4 ^M, [CC] = 10^−5 ^M, THF/H_2_O = 1/9). DLS size distribution of **d** CC (10^−4 ^M, THF/H_2_O = 1/9), **e** PNC (10^−4 ^M, THF/H_2_O = 1/9), **f** PNC/CC ([PNC] = 10^−4 ^M, [CC] = 10^−5 ^M, THF/H_2_O = 1/9). SEM images of **g** PNC (10^−4 ^M, THF/H_2_O = 3/7), **h** PNC/CC ([PNC] = 10^−4 ^M, [CC] = 10^−5 ^M, THF/H_2_O = 3/7). Green twist and red helix are cartoons to show the features of corresponding morphologies of PNC and PNC/CC respectively.

PNC and CC were synthesized via several steps including substitution and condensation reactions. Nuclear magnetic resonance and high-resolution mass spectra (HRMS) verified the successful synthesis (Supplementary Figs. 1–6). Both of the compounds were constituted by the aromatic and cholesteryl domains with different polarities that facilitate the ordered aggregation undergoing the bottom-up self-assembly^43^. Through a nanoprecipitation method initiated by solvent exchange, CC in tetrahydrofuran (THF)/water (1/9, v/v) mixture afforded a stable colloidal dispersion. Under transmission electron microscopy (TEM) observation, particles with size around 100 nm emerged (Fig. 2a). Zoomed image indicates the liposomal or vesicle formation with interior and exterior domains (Supplementary Fig. 7). Cholesteryl segments are lipids with considerable lipophilicity while the aromatic coumarin with cation is hydrophilic, which endow CC with specific amphiphilicity to give vesicles. Similarly, PNC self-assembled into vesicular particles as well. However, the size was significantly enlarged compared to that of CC, may be caused by the decreased hydrophilicity of naphthalimide segments (Fig. 2b). The diameter of PNC nanoarchitectures is up to 200–300 nm (Supplementary Fig. 8). The similar aggregation behavior and the significant structural similarity facilitated the integrated coassembly. It is thus highly expected that coassembly formed. By considering PNC as host nanoarchitecture, the intercalation of a small amount CC would dramatically change the morphology (Supplementary Fig. 9). The aspect ratio was altered and enlarged to afford short rod-like nanoarchitectures (Fig. 2c). It indicates the formation of coassembly rather than self-sorting contributed by the existence of cholesteryl domains. Dynamic light scattering (DLS) was employed to probe the size distributions (Fig. 2d–f, Supplementary Fig. 10), which show average values around 100, 200, and 70 nm respectively, in agreement with TEM images. Cholesteryl π-conjugates could respond to solvent polarity by altering the dimension, size, and supramolecular chirality^43^.

Decreasing the solvent polarity by reducing water fraction to 70 vol% witnessed the gel-like aggregates of PNC while CC retained vesicular aggregation disregarding the declined water fraction (Supplementary Fig. 11). Under TEM and scanning electron microscopy (SEM), PNC aggregates show right-handed nanohelices (Fig. 2g, Supplementary Fig. 12). These infinitely aggregated fibers with width around 100 nm suggest the impact of solvent polarity in tuning the morphology and aggregation mode of PNC. By introducing CC (10 mol%) as a dopant, the mixture retained the same morphology with identical right-handedness (Fig. 2h, Supplementary Figs. 13, 14). Coassembly of PNC and CC is thus independent to the solvent polarity. In addition, PNC in apolar decane featured a stable organogel phase with thin fibers observed by TEM, which kept stable gel phase after introducing CC (Supplementary Fig. 15).

### Energy transfer in aggregation systems

**Fig. 3:**
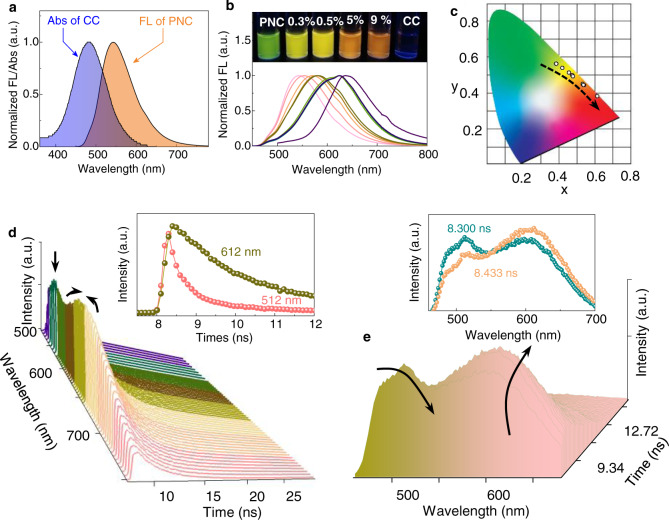
Optical properties of assemblies upon THF/H2O = 1/9 (v/v). **a** Normalized spectrum overlaps of donor emission (10^−4 ^M) and acceptor absorption (10^−4 ^M, excited at 430 nm). **b** Normalized fluorescent spectra of PNC (10^−4 ^M) with various amount of CC (from left to right: 0, 0.1, 0.3, 0.5, 0.7, 3, 5, 7, 9 mol%, excited at 430 nm) and CC (9 × 10^−6 ^M, excited at 480 nm). Insets show the pictures under 365 light irradiation (from left to right: 0, 0.3, 0.5 mol%, 5, 9 mol% and CC) and **c** corresponding CIE coordination evolution. **d** Fluorescent decay curves of PNC/CC ([CC] = 5 × 10^−7 ^M). Inset shows fluorescent decay curves of PNC/CC at emission wavelengths of 512 and 612 nm. **e** TRES of PNC/CC ([CC] = 5 × 10^−7 ^M). Inset shows TRES comparison at 8.300, 8.433 ns, respectively. [PNC] = 1 × 10^−4 ^M. (excited at 441 nm).

In aqueous self-assembly (THF/H_2_O = 1/9 v/v), the absorbance of CC and the emission of PNC are overlapped (Fig. 3a). Thus in the aggregated state, energy transfer may occur due to the structural overlap and synergistic coassembly, which allows for the short contact between donor and acceptor. PNC self-assembly in water showed green emission located at 542 nm while CC individually has very weak fluorescence due to the photo-induced electron transfer effect (Fig. 3b). However, with increasing molar equivalent of CC from 0 to 10 mol%, green-yellow-orange-red luminescent color evolution emerged with wavelength shifted to 624 nm. The gradual luminescent color conversion is initiated by the energy transfer. The energy transfer efficiency (Φ_ET_) reached 44.8 % (544 nm) at the donor/acceptor ratio of 100:5 (Φ_ET_ = 1-I_DA_, (λ_ex_ = donor)/I_D_, (λ_ex_ = donor)) (Supplementary Figs. 16, 17)^44^. Coassembly provided a lipophilic environment to CC which reduced the aqueous aggregation-induced quenching effect. The major absorption peak of PNC at 433 nm exhibited a hypochromic shift to 428 nm after the addition of CC, which might be aroused by the π–π stacking occurred between coumarin and naphthalimide moieties (Supplementary Fig. 18). Corresponding to the fluorescent spectra, the CIE coordinates were calculated (Fig. 3c). A transition from (0.3855, 0.5668) to (0.5355, 0.4473) was observed when increasing molar fraction of CC from 0 to 9 mol%, in agreement with the bulky emission colors from green to red. Fluorescent decay curves of PNC/CC mixture ([CC] = 0.5 mol%, THF/H_2_O = 1/9) at different wavelengths were shown in Fig. 3d, where the intensity at 540 nm decreased with increasing wavelength at a same decay time. Meanwhile, the fluorescent intensity at around 600 nm exhibited an increase then decrease propensity. Typically, the decay curve at 612 nm (inset of Fig. 3d) displayed an increase-then-decrease trending, in contrast to the decreased trending at around 8.5 ns. The above observations confirm the energy transfer from PNC to CC within aqueous phase nanoassemblies. In time-resolved emission spectra (TRES) indicated a decreasing tendency of green emission with increasing decay time, while the red-emission exhibited an increase-then-decrease along with increasing decay time, which can be shown by the fluorescence spectra at 8.3 and 8.433 nm (inset of Fig. 3e). TRES supports the energy migration from PNC to CC within coassemblies in nanovesicles.

**Fig. 4:**
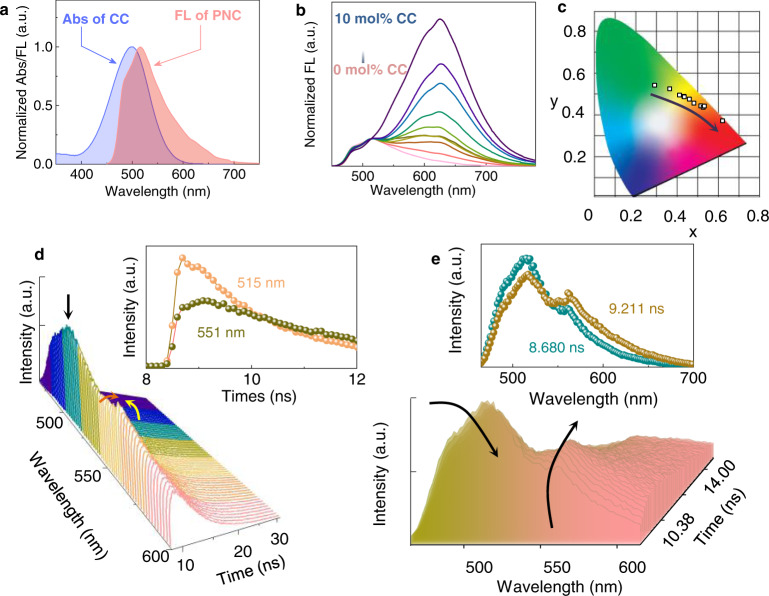
Optical properties of assemblies upon THF/H_2_O = 3/7 (v/v). **a** Normalized spectrum overlaps of donor emission (10^−4 ^M) and acceptor absorption (2 × 10^−4 ^M, excited at 430 nm). **b** Normalized fluorescent emission of PNC (2 × 10^−4 ^M) with various amount of CC (0, 0.1, 0.3, 0.5, 0.7, 1, 3, 5, 7, 10 mol%, excited at 430 nm) and **c** corresponding CIE coordination. **d** Fluorescent decay curves of PNC/CC ([CC] = 10^−6 ^M). Inset shows fluorescent decay curves of PNC/CC at emission wavelengths of 515 and 551 nm. **e** TRES of PNC/CC ([CC] = 10^−6 ^M). Inset shows TRES at 8.680 and 9.211 ns, respectively (excited at 441 nm) ([PNC] = 2 × 10^−4 ^M).

We then evaluated the energy transfer in nanohelical systems in THF/H_2_O (3/7). Compared to the dispersed vesicle phase, declining water fraction (*f*_*w*_) of PNC to 70 vol% afforded half-gel phase at the low concentration of 10^−4 ^M. The existence of large amount of organic solvent (30 vol%) aroused hypochromic shift of emission. The maximum emission peak of PNC at 516 nm gave an improved spectral overlap with the absorbance of energy transfer acceptor CC at 495 nm (Fig. 4a). It ensures the energy transfer within synergistic coassembly. By increasing molar fraction of acceptor from 0 to 10 mol%, the emission spectra exhibited pronouncedly enhanced emission at 625 nm with gradually declined pristine emission at around 516 nm, indicative of efficient energy transfer has occurred (Fig. 4b, Supplementary Fig. 19). The Φ_ET_ was calculated as 52.2% (516 nm) at the donor/acceptor ratio of 100:5. Accordingly, CIE coordinates transformed from (0.2934, 0.5443) to (0.5335, 0.4419) gradually along with the molar fraction of acceptor, realizing color evolution from green to red (Fig. 4c). It should be noted that in the presence of 10 mol% acceptor, acceptor CC remained in a molecular “free” state dispersed in PNC aggregates. Wavelength-resolved fluorescent decay curves ([CC] = 10^−6 ^M) in Fig. 4d show similar trending to vesicle system in Fig. 3. Peak at around 510 nm decreased while the peak larger than 550 nm showed increase-then-decrease trending along with decay time. It can be clearly illustrated in the inset of Fig. 4d, where the decay curve of 515 nm emission decay directly while that of 551 nm increases first then decreases. In TRES spectra, emission spectrum with the elongated decay time (9.211 ns) exhibited declined emission at donor region and enhanced emission at acceptor region, compared to the spectrum with the shorter decay time (8.680 nm) (Fig. 4e). It supports the effective energy transfer occurred between PNC and CC in half-gel system. In addition, we also evaluated the energy transfer behavior in gel system in apolar decane. The emission spectra of PNC show a spectral overlap with the absorbance of CC. However, as shown in Supplementary Fig. 20, with increasing molar fraction of acceptor CC from 0 to 1 mol%, the main emission peak keeps around 500 nm and no new emission peaks appeared, indicating energy transfer could not occur in the gel system. Due to the presence of cationic polar head of CC, the solvation effect might be declined compared with PNC, which results in self-sorted behavior rather than coassembly. Consequently, failed energy transfer occurred in the decane phase.

### Nucleophilic addition reaction

**Fig. 5:**
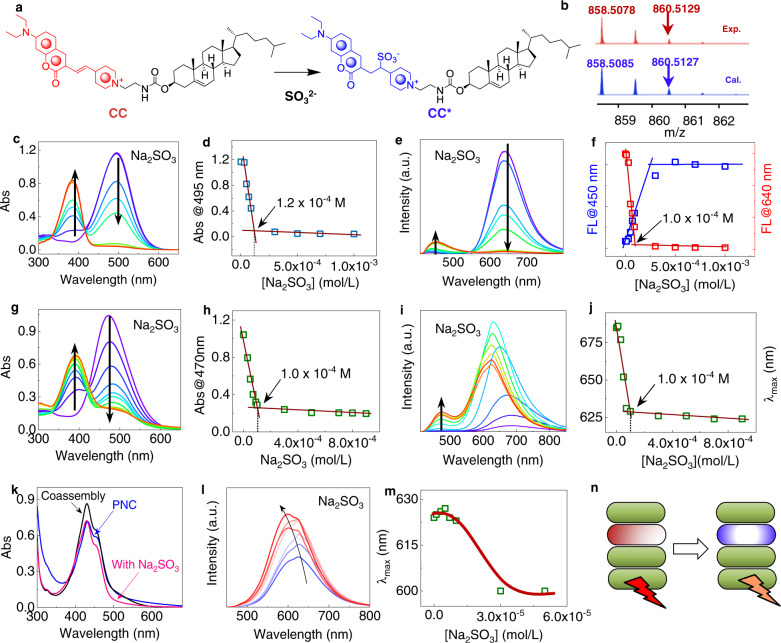
Variations of UV–Vis absorption and fluorescent emission spectra treating with Na_2_SO_3_. **a** Reaction of CC with Na_2_SO_3._ **b** Comparison of HRMS about CC treated with Na_2_SO_3_ (5 molar eq.) with stimulated profile. **c** UV–Vis absorption and **d** corresponding vibration of absorption at 495 nm of CC (10^−4 ^M) with various concentrations of Na_2_SO_3_. **e** Fluorescent emission spectra and **f** corresponding vibration of emission intensity at 450 and 640 nm of CC (10^−4 ^M) with various concentrations of Na_2_SO_3_ in THF (10 vol% water was added to dissolve Na_2_SO_3_). **g** UV–Vis absorption and **h** corresponding vibration of absorption at 470 nm of CC (10^−4 ^M) with various concentrations of Na_2_SO_3_. **i** Fluorescent emission spectra and **j** corresponding vibration of the maximum emission wavelength of CC assembly (10^−4 ^M) with various concentrations of Na_2_SO_3_ in THF/H_2_O = 1/9. **k** UV–Vis absorption comparison of PNC, CC, PNC/CC and PNC/CC/Na_2_SO_3_ assemblies. **l** Fluorescent emission spectra and **m** corresponding vibration of maximum emission wavelength of PNC/CC coassemblies ([PNC] = 10^−4 ^M, [CC] = 10^−5 ^M) with various concentration of Na_2_SO_3_ (THF/H_2_O = 1/9, excited at 380 nm). **n** A schematic model for color changing responding to the anion, where green, red and blue rods stand for PNC, CC, and CC* respectively.

CC processes a stilbene-pyridinium structure, which could react with SO_2_ derivatives^45^. Through nucleophilic addition reaction, SO_2_ derivatives like HSO_3_^−^ and SO_3_^2−^ would add to C=C bond to destruct the π-conjugated structure, resulting in the fluorescence evolution and significant hypochromic shift (Fig. 5a). After reaction with SO_3_^2−^, CC was expected to transform into CC*, which was verified by the HRMS shown in Fig. 5b. Fragment *m/z* peak at 858.5078 assigned to the [M + H]^+^ is in good agreement with the theoretical value of 858.5085, which is also isotropy-resolved, suggesting the formation of CC* after being treated with SO_3_^2−^ (Supplementary Fig. 21). In organic solvent (THF, with 10 vol% H_2_O to dissolve Na_2_SO_3_), with increasing Na_2_SO_3_ equiv., the major absorbance of CC at 495 nm gradually declined with corresponding increase at 385 nm (Fig. 5c). Such a great hypochromic shift over 100 nm is a typical phenomenon of the nucleophilic addition to stilbene^45^. Linear correlation was observed before a plateau region when plotted absorbance at 495 nm as a function of Na_2_SO_3_ concentration (Fig. 5d). The critical concentration at 1.2 × 10^−4 ^M ([CC] = 10^−4^ M) indicated a 1:1 reaction ratio, and qualitative reaction happened in diluted solution phase (reaction efficacy = 100 %). Meanwhile, in fluorescent emission spectra (Fig. 5e), red-emission at 640 nm was quenched with an appeared emission at 450 nm, which can be assigned to the coumarin moiety, confirming the reaction equation in Fig. 4a. Also the luminescence color transition point was determined as 10^−4 ^M (Fig. 5f).

Then we probed the reaction in self-assembled vesicle phase of CC (Fig. 5g–j). Aggregation normally would block the sufficient reaction or supramolecular complexation to reagents, and reaction on the surface is superior. In absorbance spectra variations, the increasing anion molar ratios resulted in an identical propensity to that of organic solvent with declined reaction efficacy (46%) (Fig. 5g, h). However, the emission spectra showed ill-defined trending with respect to intensity at red-emission regions (Fig. 5i, Supplementary Fig. 22). Such behavior is contributed by several factors including the disappearance of photo-induced electron transfer effect after anion addition and the change of aggregation modality or molecular packing. The overall emission was enhanced unexpectedly. Nevertheless, the hypochromic shift as a function of anion concentration exhibited a well-defined trending with a critical transition point at 10^−4 ^M (Fig. 5j). We also studied the effect of NaHSO_3_ on the absorption and emission of CC in self-assembled vesicle phase (Supplementary Fig. 23). Similar phenomenon was observed with Na_2_SO_3_ in absorption and emission spectra variations. By doping CC (10 mol%) into PNC vesicle phase into coassembly, the maximum absorbance at 430 nm increased slightly (Fig. 5k). After being treated with Na_2_SO_3_, absorbance decreased at 430 nm (Supplementary Fig. 24). The recovery indicates the occurrence of reaction within coassembled vesicles. Similar to the individual CC self-assembled system, the emission intensity was enhanced with specific hypochromic shift with the separation about 30 nm (Fig. 5l, m, Supplementary Fig. 25). Such hypochromic shift is associated with the restricted energy transfer due to the reduced acceptor species by Na_2_SO_3_, which would contribute to the luminescent color evolution from red to orange or yellow (Fig. 5n). In contrast to the molecular free state (THF/H_2_O = 9/1) when SO_2_ derivatives (HSO_3_^−^ or SO_3_^2−^) could be fully involved into nucleophilic addition reaction, CC in aggregated vesicle phase (Supplementary Figs. 7 and 11) showed slightly declined reaction efficacy and responsiveness to SO_2_ derivatives based on the absorption and emission spectroscopy. This phenomenon is attributed to the partial exposure of reactive groups on the surface of vesicles, and the aggregation hinders full contact of reagents. In spite of the unfavorable factor, nucleophilic addition reaction occurred sufficiently to arouse emission variation and luminescent color conversion in aggregated state.

Decreasing the amount of water (*f*_*w*_ = 70%), absorption also sharply decreased around 500 nm and new peak appeared at 400 nm after being treated with Na_2_SO_3_. Absorption at 500 nm from π-conjugated structure could not completely disappear even treated with 5 equiv. anion. Similar phenomenon was observed in fluorescent emission spectra. As show in Supplementary Fig. 26, after treating with 5 equiv. anion, aggregates simultaneously emitted fluorescence at 469 and 632 nm which was completely disappeared at 632 nm in dispersive state (THF/H_2_O = 9/1), indicating the addition reaction just reached an equilibrium in the aggregative state, which may be attributed to the compact molecular stacking. UV–vis absorption at 500 nm and fluorescent emission around 632 nm only showed slightly decrease, and no obvious new peaks appeared after being treated with NaHSO_3_. Doping NaHSO_3_ into PNC/CC ([PNC] = 2 × 10^−4^, [CC] = 2 × 10^−5^]) coassembly in THF/H_2_O (3/7) system, the emission at 516 nm corresponding the emission of PNC exhibited increasing trending, indicating the reaction occurred within coassembled system. Meanwhile, the emission at 632 nm supported that the degree of reaction was limited (Supplementary Fig. 27). HSO_3_^−^ is difficult to react with C=C bond in THF/H_2_O = 3/7, which might be contributed by several factors including the nucleophilic ability of HSO_3_^−^ and relative compact molecular packing. It should be noted that failed energy transfer occurred in the decane phase. Thus, it is meaningless to investigate the SO_2_ responsiveness. In addition, the apolar nature of decane does not allow for the dissolution of the employed anions (HSO_3_^−^ or SO_3_^2−^). On top of the above facts, SO_2_ responsiveness could not be realized in the decane system.

### Chiroptical properties

**Fig. 6:**
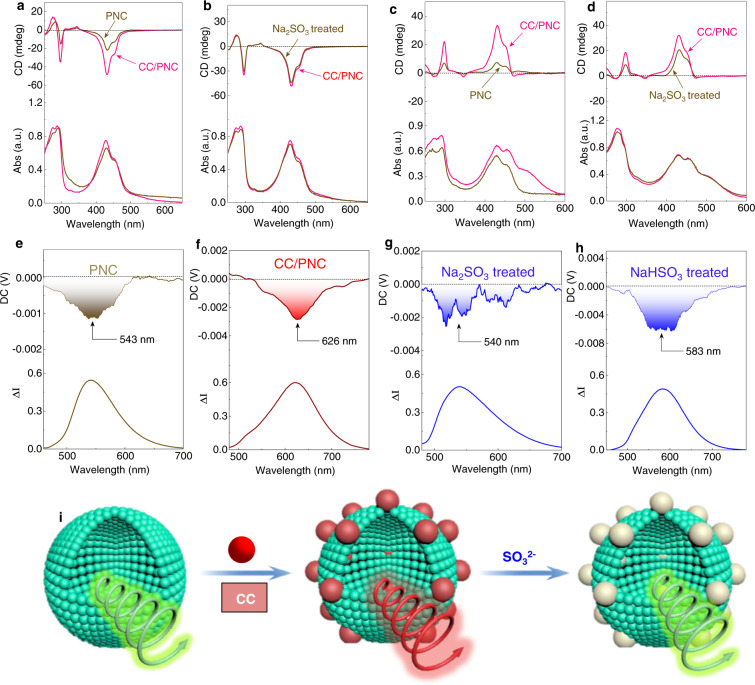
Influence of coassembly and anions to CD and CPL spectra. Comparison of CD and corresponding UV–Vis absorption spectra of **a** PNC and PNC/CC assemblies and **b** PNC/CC and PNC/CC/Na_2_SO_3_ assemblies (THF/H_2_O = 1/9). Comparison of CD and corresponding UV–Vis absorption spectra of **c** PNC and PNC/CC assemblies and **d** PNC/CC and PNC/CC/NaHSO_3_ assemblies (THF/H_2_O = 3/7). CPL spectra and corresponding fluorescent emission of **e** PNC **f** PNC/CC **g** PNC/CC/Na_2_SO_3_ **h** PNC/CC/NaHSO_3_ (film). **i** Representation of CPL variation with different additives. Blue, red and gray particles represent PNC, CC and CC* respectively. ([PNC] = 10^−4 ^M, [CC] = 10^−5 ^M, [Na_2_SO_3_] = 5 × 10^−5 ^M, [NaHSO_3_] = 5 × 10^−5 ^M, THF/H_2_O = 1/9, excited at 430 nm).

In vesicle phase, PNC individual self-assembly afforded negative Cotton effects at around 430 and 280 nm corresponding to the absorbance peaks of phthalimide. The addition of CC (10 mol%) slightly increased the absorbance as well as the Cotton effects (Fig. 6a). Dissymmetry g-factor at 430 nm was enhanced from −1.0 × 10^−3^ to −1.9 × 10^−3^ after binding with CC, contributed by the synergistic effect (Fig. 6a, Supplementary Fig. 28). After being treated with Na_2_SO_3_, only tiny decrease in CD and absorbance were observed (Fig. 6b, Supplementary Fig. 29). Chirality at ground state reflected on Cotton effects could barely influenced by the structural evolution. In half-gel state (THF/H_2_O = 3/7), positive Cotton effects at 430 and 280 nm gave a mirror curve. It suggests decreasing water fraction caused a supramolecular chirality inversion (Fig. 6c). According to the exciton-chirality theory, the positive Cotton effect corresponds to the right-handed helical sense of chromophores, which is in good agreement with the right-handed helices observed by SEM (Fig. 2). Thus, the helical sense in vesicle membranes might follow a left-handedness. Similarly, after introducing CC, the g-factor of absorbance was also enhanced sharply, confirming the synergistic coassembly occurred independently of the solvent polarity (Supplementary Fig. 30). After being treated with Na_2_SO_3_, the Cotton effect was declined (Fig. 6d), indicative of the partial destruction of coassembly. The helical arrangement in vesicles enabled the CPL activity. Negative CPL signals at 540 and 670 nm were generated in individual self-assemblies of PNC and CC respectively, with dissymmetry g-factors (g_lum_) of −2.0 × 10^−3^ and −4.0 × 10^−3^ respectively. As shown in Fig. 6f and Supplementary Fig. 31, the doping of CC into PNC vesicles with increasing molar fraction witnessed the red-shifted CPL at 574 nm (0.7 mol%, g_lum_ = −3.0 × 10^−3^), 592 nm (3 mol%, g_lum_ = −5.0 × 10^−3^) and 626 nm (10 mol%, g_lum_ = −5.0 × 10^−3^). Energy transfer between PNC and CC allowed for the CPL color evolution from green to red depending on the fraction of CC. After being treated with Na_2_SO_3_, fluorescence was recovered to 540 nm with hypochromic-shifted CPL (Fig. 6g). In contrast, NaHSO_3_ under a similar condition only partially recover the fluorescence and CPL (Fig. 6h) probably due to the weak oxidative capability. Compared with PNC/CC coassembly, no significant changes were observed in morphology from TEM images after treating with anion (Supplementary Fig. 32). In half-gel phase, due to the structure-persistent fiber structures, partial CPL recovery was also observed, giving rise to orange CPL after being treated by SO_2_ derivatives (Supplementary Fig. 33). The intrinsic helical structures were formed by PNC individually in a low fraction of water/THF mixture (3:7, v/v, Fig. 2g). The participation of CC would barely change the helical topology such as helicity and handedness due to its low molar fraction (10 mol%, Fig. 2g, h). Treated by sulfite anion, CC transformed into CC*. CC is a cationic molecule (pyridinium), while CC* is zwitterionic after appended with sulfite. The zwitterionic CC* may be appended with better hydrophilicity. Normally, the variation of amphiphilicity shall influence the self-assembled morphology. However, in the present system, CC or CC* as an energy transfer acceptor, only 10 mol% was used. In addition, due to the limited exposed reactive coumarin groups on the surface of helices, it is assumed that partial CC within coassembled helical structures were transformed into CC*. Thus, apparent morphological evolution was not expected after being treated by sulfite anion. Experimental results confirmed the above assumption. As shown in Supplementary Fig. 34, TEM and SEM images of PNC/CC* show the presence of helical structures which are almost identical (with respect to handedness, size and lateral length) to the pristine PNC/CC coassembly. We realized anion-responsive CPL using a fluorescent sensor as a chiral building block (Fig. 6i). It is worth noting that, in the SEM and TEM samples of PNC/CC*, a certain amount of Na_2_SO_3_ was added in the mixture of THF/H_2_O mixture (*f*_*w*_ = 70 vol%). In order to fully transform CC to CC*, excess Na_2_SO_3_ (10^−4 ^M) were added. However, the unreacted Na_2_SO_3_ would majorly dissolved in aqueous media, which means the influence on the aggregated helices could be negligible. In addition, the resultant CC* is a zwitterionic building unit that normally shows enhanced resistance to ionic strength. Through TEM and SEM observation, no crystalline salt structures were found, suggesting the absence of salts in TEM and SEM samples.

For the luminophores-conjugated with chiral centers, self-assembly shall transfer chirality to luminophores. The helical packing of luminophores with certain handedness arouses asymmetrical arrangement of electronic and magnetic dipole moments to afford chiroptical properties including CD and CPL signals. By this reason, CC gave rise to negative CPL centered at around 670 nm independent to the solvent ratios (Supplementary Figs. 31, 33). The main factor for the emergence of CPL in the individual CC self-assemblies is the chiral packing of luminophores induced by cholesteryl groups. For the coassemblies with PNC, energy transfer shall occur between PNC and CC. The transferred energy from PNC contains both natural and polarized luminescence, which further excite CC to afford CPL. After coassembly with PNC, CC would adapt to the helical packing of PNC as a guest species (10 mol%). Therefore, the main factor in the CPL of CC in coassemblies originates from the helical packing modality of PNC and transferred energy from donor (PNC). After being treated with Na_2_SO_3_, the energy transfer was blocked (especially in vesicle phase), and the CPL of CC* was diminished due to the nucleophilic addition reaction.

### Stacking model

**Fig. 7:**
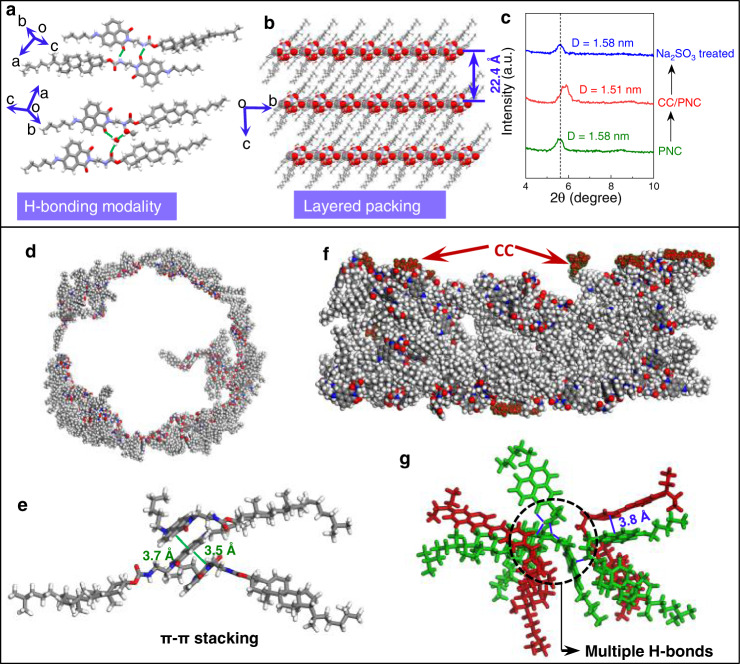
Stacking model of assemblies. **a**, **b** Hydrogen bonding modality as well as packing of PNC in X-ray structures. If not specifically noted, C, H, O, and N atoms are marked in gray, white, red, and blue respectively. **c** Small angel XRD pattern comparison of PNC, PNC/CC coassembly and Na_2_SO_3_ treated PNC/CC coassembly. **d**, **e** MD simulation results (50 ns) of PNC as well as the detailed π-π stacking interactions between naphthlimides. **f** The corresponding MD simulation results of PNC/CC coassembly. Dark red clusters represent coumarin moieties of CC. **g** Specific packing mode of CC/PNC where green and red stand for PNC and CC respectively for clarity. Blue solid lines represent weak interactions.

In order to give insights into the molecular packing and coassembly behaviors, a single crystal of PNC was cultured in methanol/dichloromethane mixture (Fig. 7a). Dimer was found linked by duplex hydrogen bonds between amide and imide carbonyl group, which enables the close packing of the alkyl chain and cholesteryl segment. The dimers are further linked by the entrapped water and methanol via hydrogen bonds into hydrogen-bonded networks. In packing mode, the polar (amide and imide) and apolar (alkyl and cholesteryl) domains constitute into lamellar structure (Fig. 7b). The interlayer distance was determined as 22.4 Å. As vesicle structures generate no ordered patterns, we deposited half-gel phase with *f*_*w*_ = 70 vol% to thin-film small angle XRD test (Fig. 7c). A diffraction peak at 5.6° with a d-spacing of 1.58 nm shifted to 5.86° with a distance of 1.51 nm after incorporated with CC (10 mol%), indicating the active participation of CC into PNC helical fibers rather than physical mixing. After being treated with Na_2_SO_3_, the peak and distance recovered to the original location, in consistent to the fluorescence recovery. Then we employed molecular dynamic (MD) simulation to explore the self-assembly and packing in individual and coassembly systems. MD simulations for all systems were carried out for 50 ns with a time step of 0.001 ps per integration step under the ensemble conditions of *T* = 298 K. All simulations were visualized using VMD program. Four models including PNC (THF/H_2_O = 1/9 and 3/7) and PNC/CC (THF/H_2_O = 1/9 and 3/7) were prepared. The simulation box sizes were fixed as 20 × 10 × 10 nm^3^. The number of PNC, CC, and total numbers of solvent molecules were fixed on 100, 10, and 30000, respectively. The free molecular arrangement of PNC in THF/H_2_O (1/9) in the original box transformed into a toroidal structure after 50 ns optimization (Fig. 7d), which was not observed in system with THF/H_2_O (3/7) (Supplementary Fig. 35). The diameter of the toroid was determined around 15 nm, much smaller than the measured vesicle particles. Similar to the packing in crystal lattice, packing of PNC was directed by polarity, whereby π–π stacking between naphthalimides dominated (Fig. 7e). In the presence of 10 mol% CC, optimization for 50 ns generated a two-dimensional layer structure (Fig. 7f), where the coumarin segments of CC packed on the rim due to the hydrophilicity. The coassembly between CC and PNC was driven by hydrogen bonds and π–π stacking, which is illustrated in the specific aggregation cluster extracted from MD results (Fig. 7g). Hydrogen bonds occur between amide/imide groups and aromatic stacking between coumarin and naphthalimides facilitate the synergistic coassembly, allowing for the efficient energy immigration.

## Discussion

In summary, cholestryl-naphthalimide and coumarin derivatives were designed and synthesized, which could coassembled into vesicles and nanohelices due to the structural similarity. Coassembly enabled the efficient energy transfer to show gradual bathochromic fluorescence shift with CPL evolution, disregarding the nanoarchitectures. CC could respond to SO_2_ derivatives via addition interaction, which would dramatically change the absorbance and emission properties of CC, which shall block the efficient energy transfer process, and aroused hypochromic shift in both luminescence and CPL. Using the coassembly and fluorescent chemosensor, we in the first time, fabricated a switchable CPL that responding to SO_2_ derivatives. Combination of chemosensors and supramolecular chirality would spark the design of functional luminescent systems with abundant chiroptical activities, which may be potentially used in chiral sensing and probing.

## Methods

### Self-assembly of PNC and PNC/CC

PNC and CC were dissolved into THF as 10^−2 ^M and 10^−3 ^M stock solutions, respectively. PNC stock solution (10 μL) was injected into 90 μL THF, and then 900 μL deionized (DI) water was added. After aging for 8 h, assemblies were obtained (THF/H_2_O = 1/9, v/v). A series of PNC/CC coassemblies were prepared via mixing various amounts of CC stock solution with PNC stock solution. For example, CC stock solution (10 μL) was mixed with 10 μL PNC stock solution. Then, THF (80 μL) and 900 μL DI water were successively added into the solution to fixing the ratio of THF and H_2_O ([PNC] = 10^−4 ^M, [CC] = 10^−5 ^M, THF/H_2_O = 1/9). We also prepared the assemblies with THF/H_2_O = 3/7. Firstly, 10 μL CC stock solution was mixed with 10 μL PNC stock solution. Then, 280 μL THF and 700 μL DI water were successively added into the mixture (THF/H_2_O = 3/7). Na_2_SO_3_ and NaHSO_3_ were introduced via dissolving into water as 100 mM stock solutions.

### MD simulation of PNC and PNC/CC

The native structures of PNC, CC, H_2_O, and THF were built from the GaussView6.0 program. The obtained configurations were initially optimized by Hartree-Fork method. The 6-31 G(d) basis set was employed in Gaussian 16 program. The Antechamber program was used to fit the restrained electrostatic potential (RESP) charge, and then the general Amber force field (GAFF) was adopted to parameterize the bonded interaction of PNC, CC, H_2_O, and THF for subsequent MD simulations. All MD simulations were implemented with the GROMACS 2020 program. All molecules were coupled to temperature in 298 K. The cut-off distance for non-bonded interactions was set to 1 nm. Energy minimization was conducted using the steepest descent algorithm before performing dynamic simulations. MD simulations for all systems were carried out for 50 ns with a time step of 0.001 ps per integration step under the ensemble conditions of *T* = 298 K. All simulations were visualized using VMD program. Four models including PNC (THF/H_2_O = 1/9 and 3/7) and PNC/CC (THF/H_2_O = 1/9 and 3/7) were prepared. The simulation box sizes were fixed as 20 × 10 × 10 nm^3^. The number of PNC, CC, and total number of solvent molecules were fixed on 100, 10, and 30,000, respectively.

## Supplementary information

Supplementary Information

## Data Availability

All data generated or analyzed during this study are included in this published article (and its supplementary information files). The X-ray crystallographic coordinates for structures reported in this article have been deposited at the Cambridge Crystallographic Data Centre, under deposition number CCDC: 2102083.
