# Supplementary material for: A chemosensor-based chiral coassembly with switchable circularly polarized luminescence
Source: Nat Commun. 2021 Nov 3;12:6320. doi: 10.1038/s41467-021-26700-2 (PMC8566482; doi:10.1038/s41467-021-26700-2)
Supplement: Supplementary file 1 — Supplementary Information [file 41467_2021_26700_MOESM1_ESM.pdf]

A chemosensor-based chiral coassembly with switchable circularly polarized luminescence

Qiuhong Cheng, Aiyou Hao and Pengyao Xing*

Key Laboratory of Colloid and Interface Chemistry of Ministry of Education and School of Chemistry and Chemical Engineering, Shandong University, Jinan 250100, People's Republic of China.

Email: xingpengyao@sdu.edu.cn

Methods section

Materials

All solvents were purchased from Guoyao Chemical Reagent Co. Ltd. (Shanghai). 3-methylpyridine (Beijing, Ouhe, 98 %), n-pentylamine (Beijin, Mreda, 99 %). Both chemicals and solvents were used without further purification. Water is deionized (DI) water.

Characterizations

^1H NMR and ^{13}C NMR spectra were measured on a Bruker AVANCE III HD 400 MHz spectrometer (USA) at room temperature with tetramethylsilane (TMS) as reference. Dimethyl sulfoxide-d (DMSO-d_6) and chloroform-d ($CDCl_3$) were used as solvent. High-resolution mass spectrometry (HR-MS) was performed via Agilent Q-TOF 6510 MS spectrometer (USA). Transmission electron microscope (TEM) images were measured by a HITACHI JEM-100CX II electron microscope (Japan). The samples for TEM detection were dropped in the copper grid and air-dried. Scanning electron microscope (SEM) images were measured by a Zeiss scanning electron microscope (Germany). The samples for SEM detection were dropped in the silicon pellet, dried and then sprayed by the gold before detection. Powder X-ray diffraction (XRD) patterns were collected on a Rigaku SmartLab polycrystall X-ray diffraction (Japan) with Cu Kα radiation (λ = 0.15406 nm, voltage 45 KV, current 200 mA, power 9 KW). The samples were casted onto cover glasses (18 mm × 18 mm) and dried to form thin films. FL and UV-Vis spectra were recorded via RF-6000 and UV-1900 from SHIMADZU (Japan), respectively. Time-resolved fluorescence spectroscopy were measured on a

FLS920 Steady State and Transient State Fluorescence Spectrometer (Edinburgh Instruments). Circular dichroism (CD) and circularly polarized luminescence (CPL) spectra were measured with Applied Photophysics Chirascan V100 (UK). Aggregations were centrifuged and dried before testing for CPL. The average diameter of aggregation was recorded by DLS measurement with NanoZS of Malvern Panalytical (UK).

Synthesis of Compound CC

Pyridine (18.0 mg, 2.00 mmol) was added into $CHCN_3$ (5.00 mL) solution of compound **1**[1]. The mixture was heated to 80 °C for 12 h under N_2 protection. After cooling down to room temperature, excess solvent was poured out and obtained crude white product.[2] Crude material (3.80 g) and compound **2**[3] (1.23 g, 5.00 mmol) were dissolved in ethanol (5.00 mL) and refluxed for 2 h. The reaction solution was washed three times with ethyl acetate and recrystallized with ethyl acetate/diethyl ether for several times until getting pure white solid CC (**3**) (2.56 g, 2.99 mmol). Yield: 59.7%. mp: 172-174 °C. ^1H NMR (400 MHz, DMSO-d_6, 298 K) δ 8.79 (d, 2H), 8.20 (d, 2H), 8.16 (s, 1H), 7.83 (d, 1H), 7.69 (d, 1H), 7.54 (d, 1H), 7.37 (m, 1H), 5.01 (s, 1H), 4.55 (m, 2H), 3.49 (m, 6H), 0.49 (s, 3H). ^{13}C NMR (126 MHz, DMSO-d_6, 298 K) δ 161.52, 158.05, 156.38, 154.38, 152.53, 146.71, 145.07, 140.16, 137.96, 132.48, 124.32, 123.04, 122.53, 114.88, 111.41, 108.88, 97.43, 73.77, 60.28, 56.30, 55.94, 55.58, 49.71, 46.90, 46.29, 42.82, 41.49, 39.32, 38.89, 37.58, 36.41, 36.01, 35.56, 31.65, 31.04, 28.19, 27.85, 24.64, 24.01, 23.63, 23.13, 22.86, 20.90, 19.37, 19.00, 13.35, 12.05. HRMS (m/z): $[M]^+$ calcd for $C_{50}H_{70}N_3O_4^+$ 776.5361; found, 776.5362.

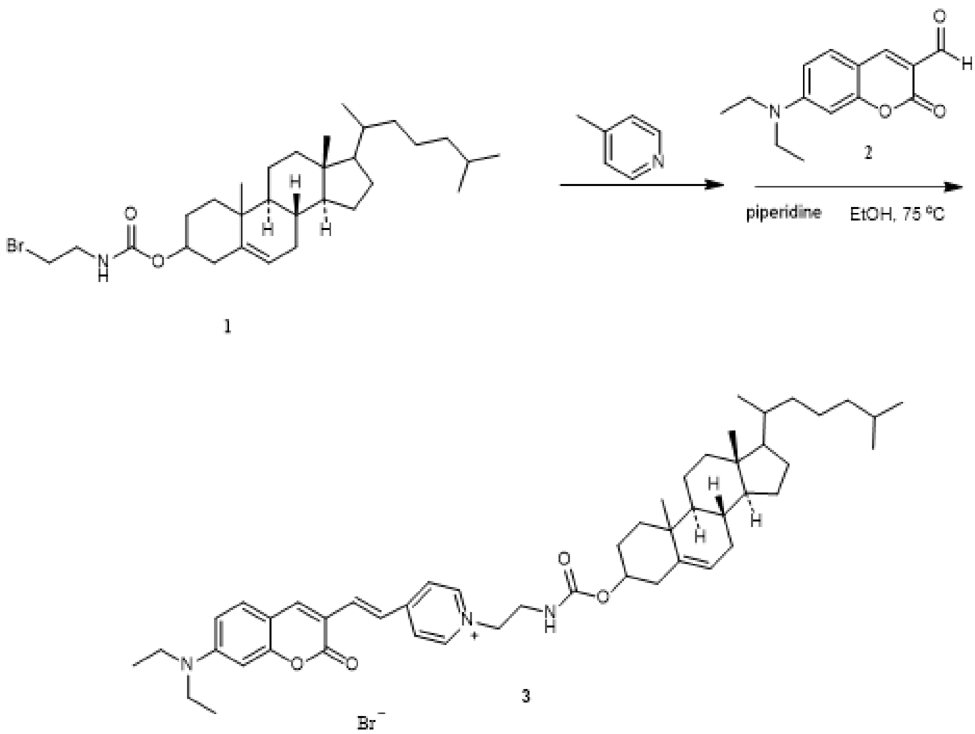

Supplementary equation (1). Synthetic route for compounds CC.

Preparation of compound PNC

Compound **4**[4] (360 mg, 0.500 mmol) and pentylamine (436 mg, 5.00 mM) were added into DMSO (30.0 mL) which was stirred upon 100 °C with N_2 protection for 8h. After cooling the mixture to room temperature, the mixture was poured into ice water (200 mL) and extracted by ethyl acetate three times. The organic layer was dried by anhydrous Na_2SO_4. After removing most solvent, the residue was subjected to column chromatography (DCM/MeOH from 1000/1 to 100/1) to afford the pure compound PNC (**5**) (250 mg, 0.339 mmol). Yield: 33.9%. mp: 183-185 ºC. PNC is orange solid. ^{1}H NMR (400 MHz, $CDCl_3$-d, 298 K) δ = 8.51 (d, 1H), 8.39 (d, 1H), 8.03 (s, 1H), 7.60-7.52 (m, 1H), 6.70 (s, 1H),5.17 (s, 1H), 4.29 (t, 3H), 3.50-3.32 (m, 4H), 2.12 (m, 2H), 1.94-0.78 (m, 52H), 0.59 (s, 3H). ^{13}C NMR (101 MHz, $CDCl_3$-d) δ = 165.09, 164.56, 156.35, 139.98, 134.79, 131.40, 129.96, 126.05, 124.75, 122.89, 122.20, 120.23, 74.13, 56.68, 56.13, 49.97, 42.30, 40.68, 39.74, 39.52, 39.37, 38.41, 36.96, 36.51, 36.19, 35.80, 31.86, 29.31, 28.65, 28.23, 28.02, 24.28, 23.83, 22.82, 22.56, 22.46, 21.02, 19.32, 18.71, 14.01, 11.84. HRMS (m/z): $[M+H]^+$ calcd. for $C_{47}H_{68}N_3O_4$, 738.5210; found, 738.5164.

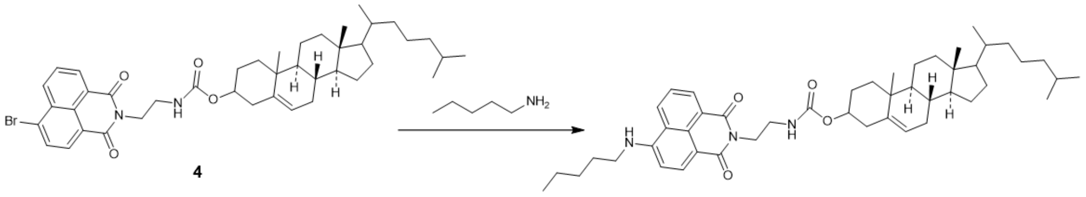

4

5

Supplementary Equation (2). Synthetic route of PNC.

X-ray crystallography

The single crystal of PNC (mp: 183-185 ºC) was cultured in methanol/dichloromethane mixture. Single crystals of PNC of appropriate dimensions were chosen under an optical microscope and quickly coated with high vacuum grease (Dow Corning Corporation) to prevent decomposition. Crystals were mounted on CryoLoop™ loop, and the cell parameters and intensity data were recorded on a Rigaku Oxford Diffraction XtaLAB Synergy-S diffractometer equipped with a HyPix-6000HE Hybrid Photon Counting (HPC) detector operating in shutterless mode and an Oxford Cryosystems Cryostream 800 Plus using Cu Kα (λ = 1.54184 Å) radiation from a PhotonJet micro-focus X-ray Source at 173 K. Data were processed using the CrystAlisPro software suite. This structure was solved using the charge-flipping algorithm, as implemented in the program SUPERFLIP and refined by full-matrix least-squares techniques against F_o^2 using the SHELXL program through the OLEX2 interface. Hydrogen atoms at carbon were placed in calculated positions and refined isotropically with a riding model. Appropriate restraints or constraints were applied to the geometry and the atomic displacement parameters of the atoms in the cluster. This structure was examined using the Addsym subroutine of PLATON to ensure that no additional symmetry could be applied to the models. The crystal data were showed in below table.

Supplementary Table 1. Crystal data of PNC

Deposition Number	2102083
Formula	C47 H67 N3 O4
Temperature (K)	173
Wavelength	1.54184Å
Crystal system	triclinic

Space group	P 1
a,b,c/Å	8.75063(18) 11.7645(3) 22.4516(5)
V, Å^3	2281.15
Cell angles	81.7980(17) 85.6733(17) 89.4848(17)
Z, Z’	Z: 1 Z': 0
R-factor (%)	7.17

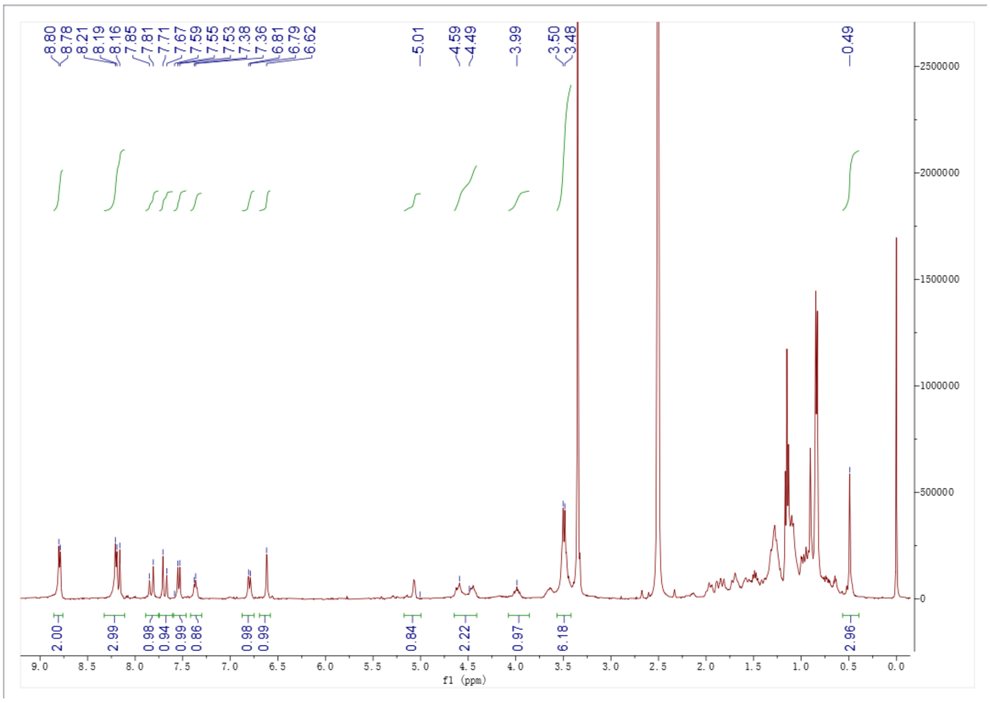

Supplementary Fig. 1. ^{1}H NMR spectrum of compound CC (**3**).

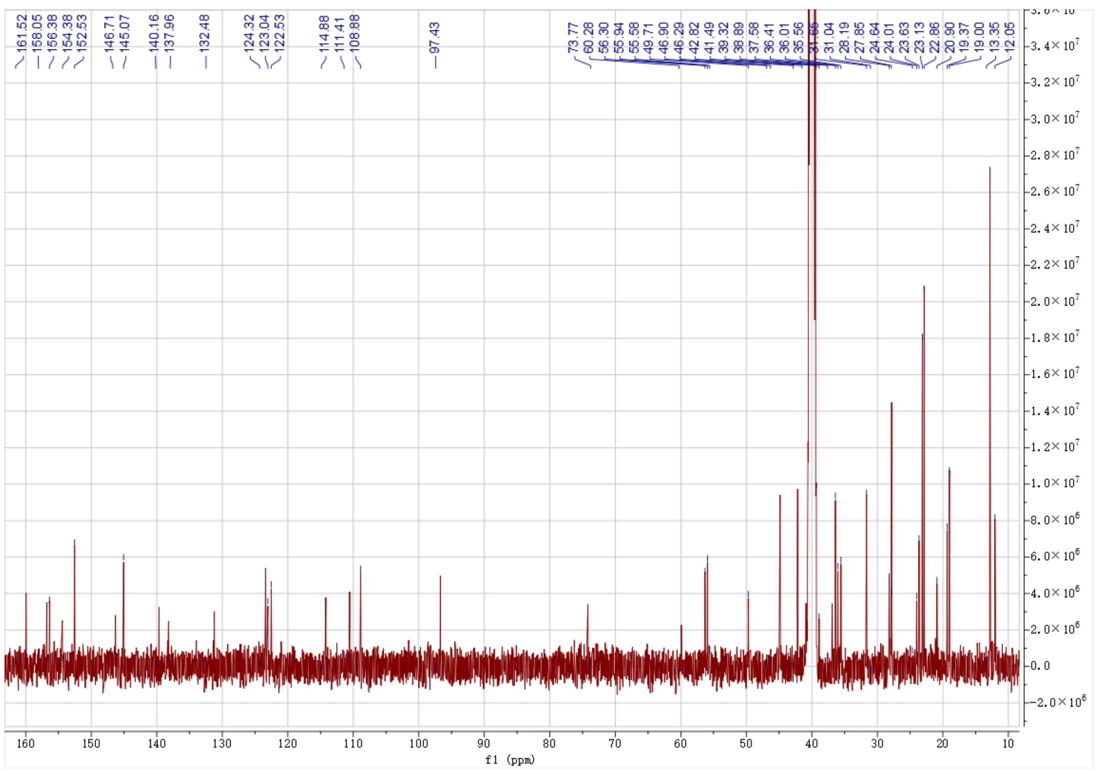

Supplementary Fig. 2. ^{13}C NMR spectrum of compound CC (**3**).

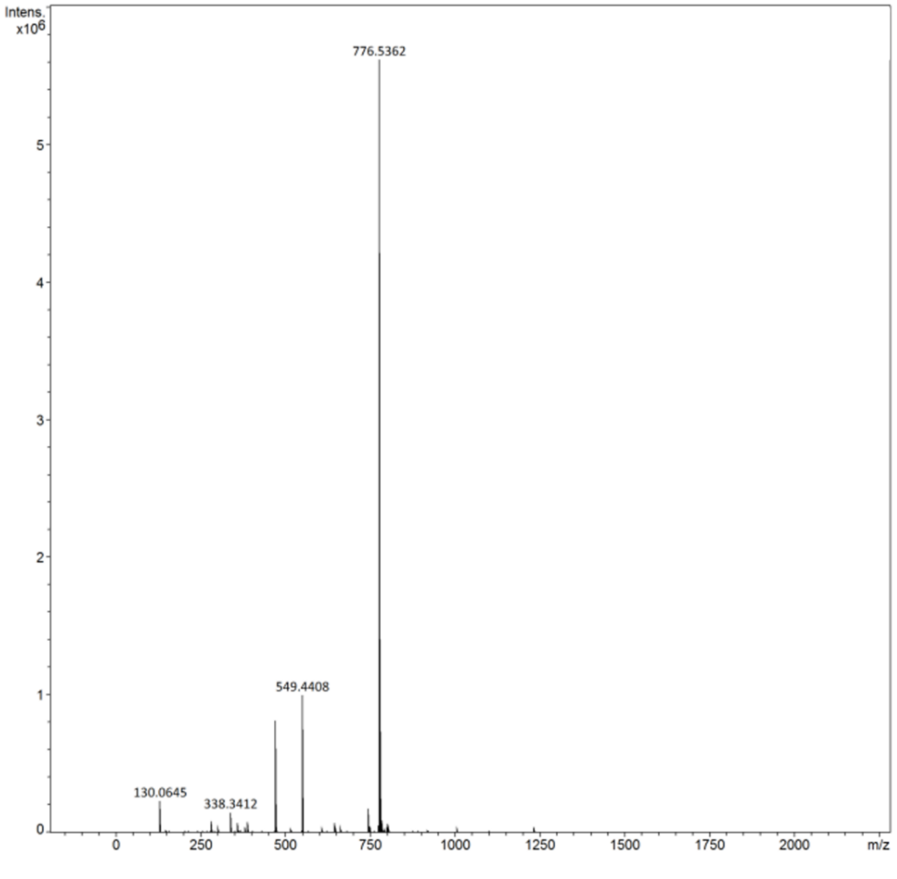

Supplementary Fig. 3. HR-MS spectrum of compound CC (**3**).

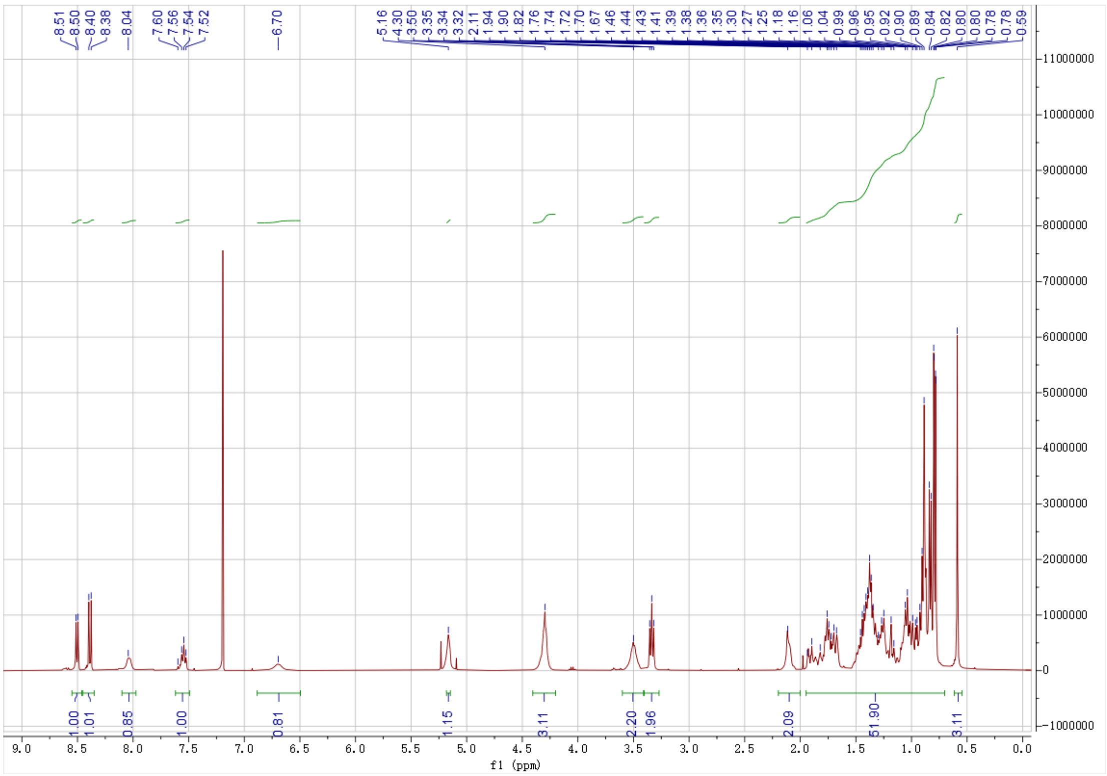

Supplementary Fig. 4. ^{1}H NMR spectrum of compound PNC.

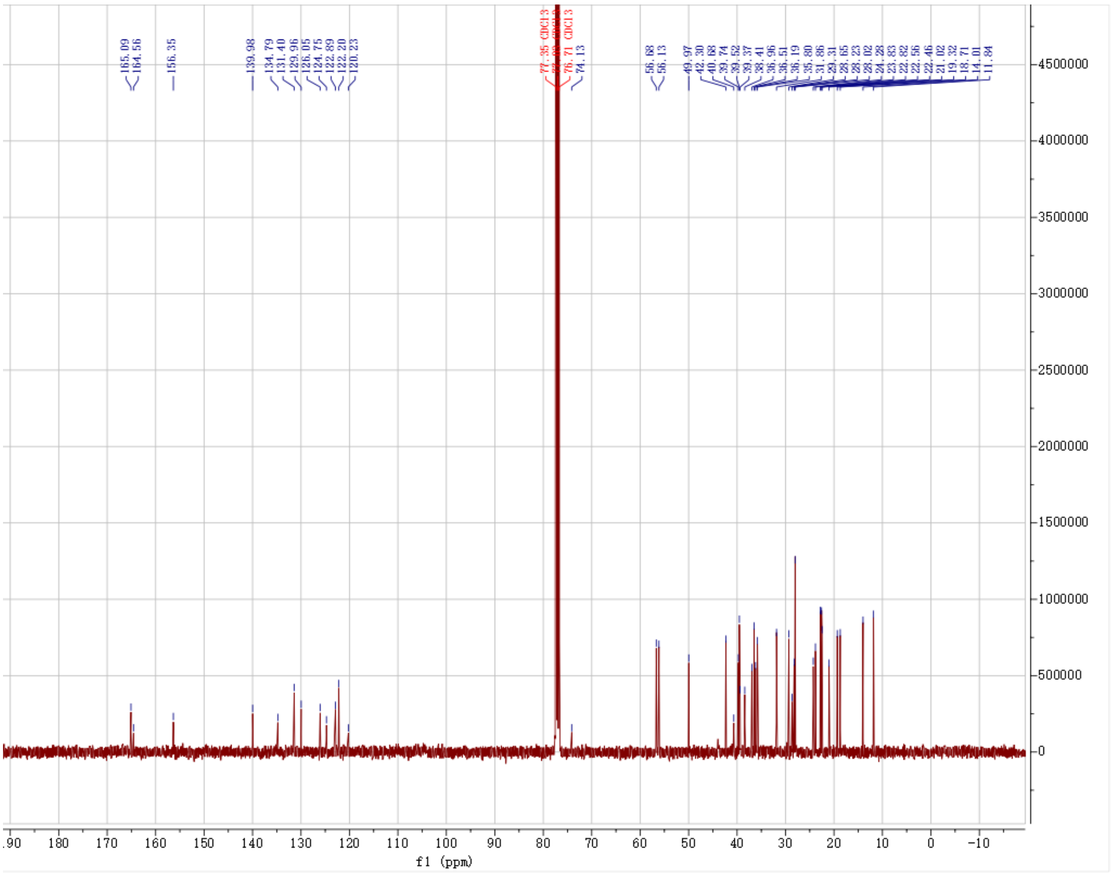

Supplementary Fig. 5. ^{13}C NMR spectrum of compound PNC.

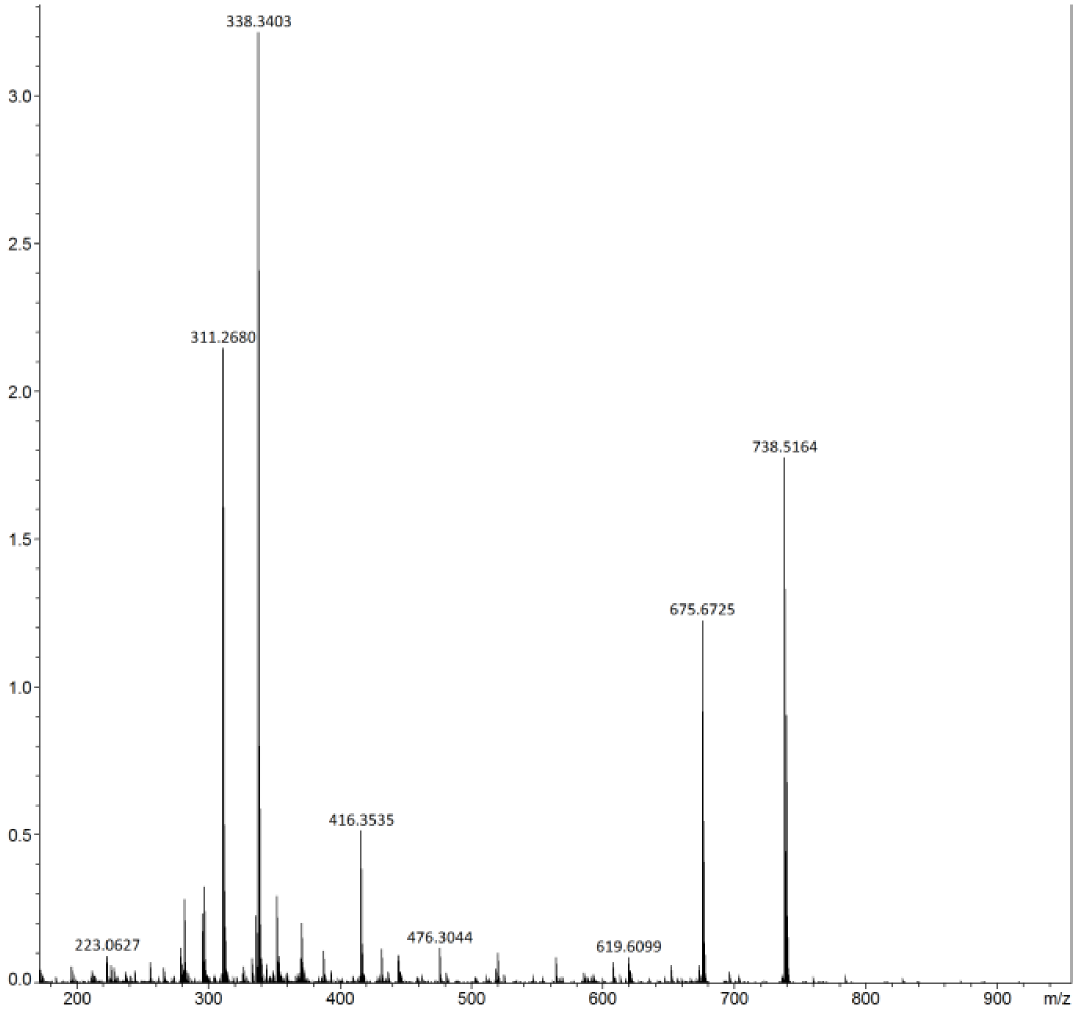

Supplementary Fig. 6. HR-MS spectrum of compound PNC.

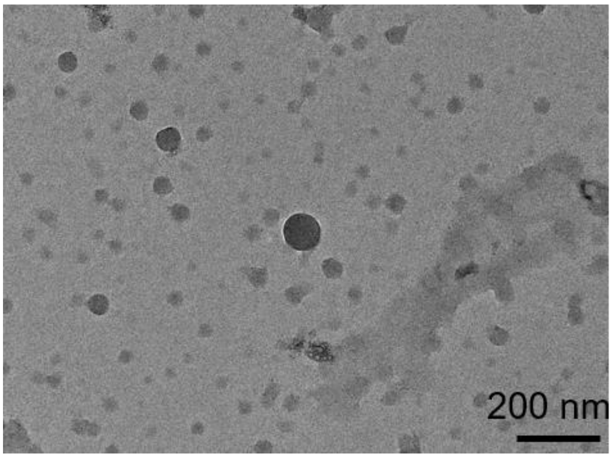

Supplementary Fig. 7. TEM image of CC assemblies (10^{-4} M, THF/H_2O = 1/9).

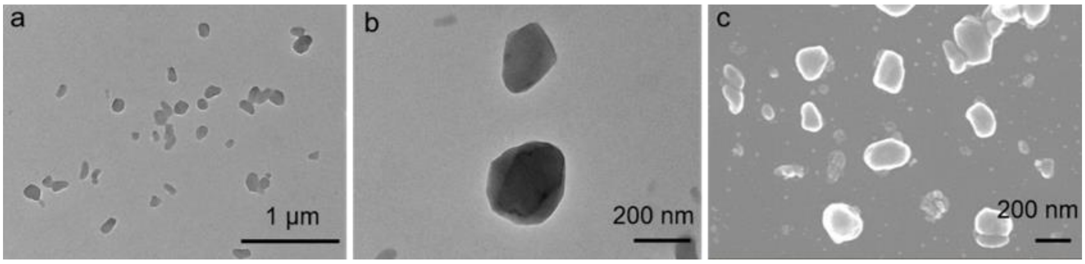

Supplementary Fig. 8. TEM **a, b** and SEM **c** images of PNC assemblies (10^{-4} M THF/H_2O = 1/9).

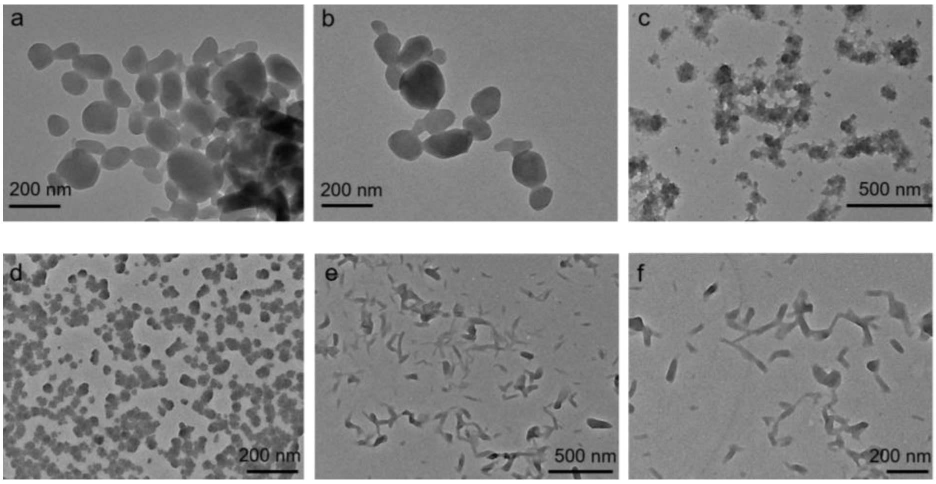

Supplementary Fig. 9. TEM images of PNC (10^{-4} M) with various amount of CC. **a, b** 0.3 mol%, **c** 0.7 mol%, **d** 3 mol%, **e, f** 10 mol% (THF/H_2O = 1/9).

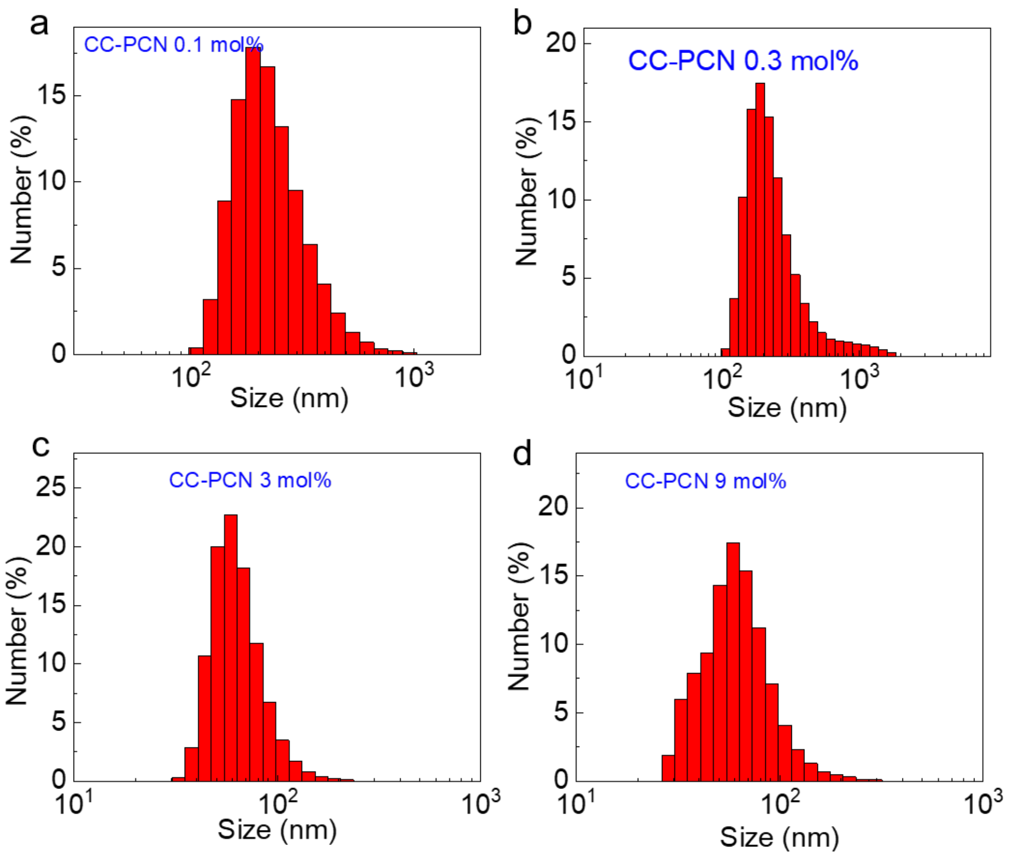

Supplementary Fig. 10. DLS of PNC (10^{-4} M) with various amount of CC. **a** 0.1 mol%, **b** 0.3 mol%, **c** 3 mol%, **d** 9 mol% (THF/H_2O = 1/9).

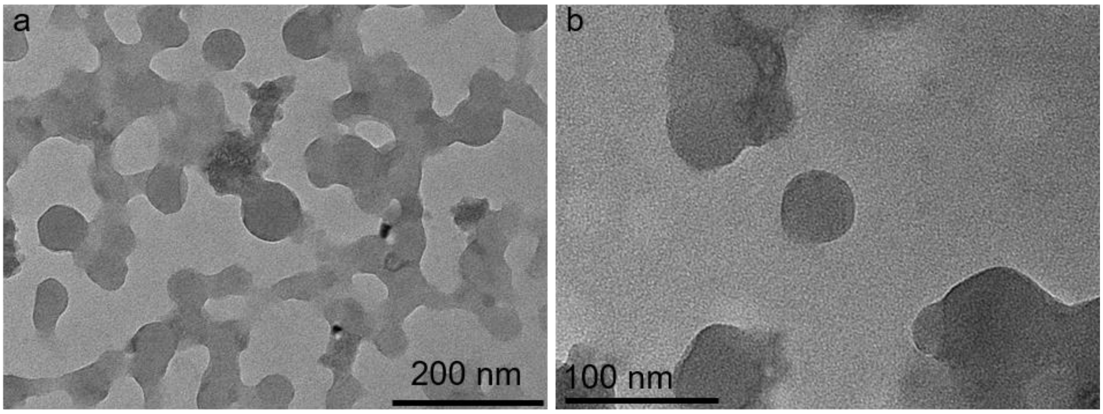

Supplementary Fig. 11. TEM images of CC self-assemblies (10^{-4} M, THF/H_2O = 3/7).

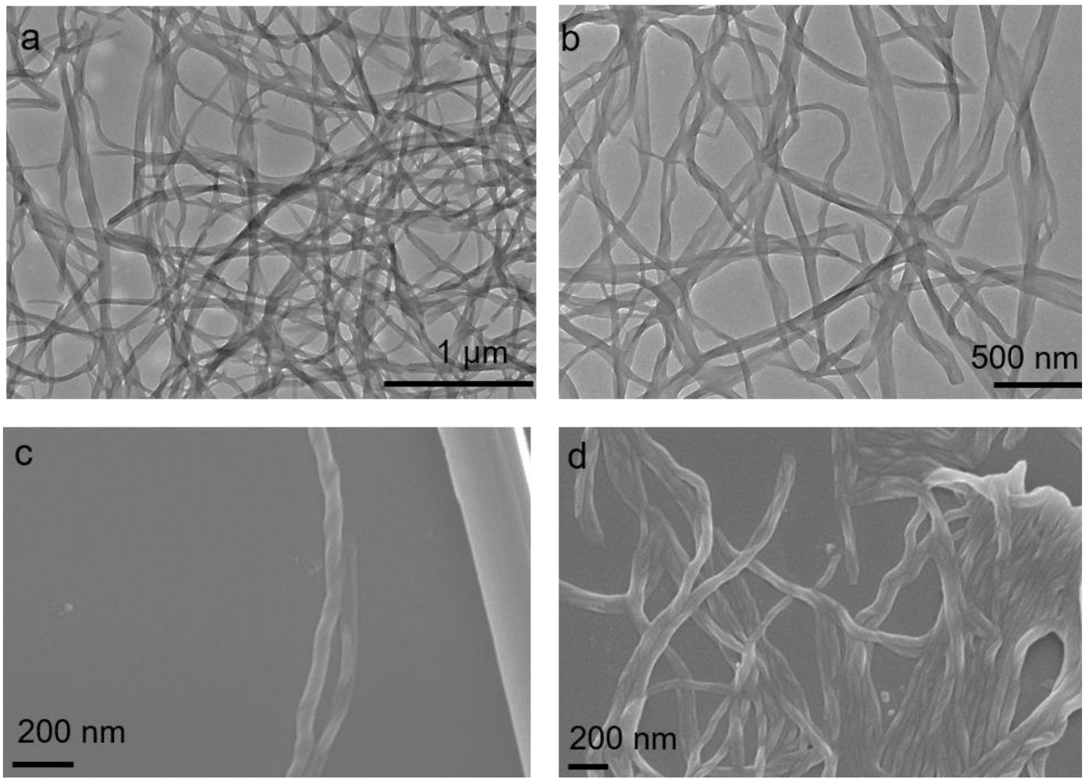

Supplementary Fig. 12. TEM **a, b** and SEM **c, d** images of PNC assemblies (10^{-4}, THF/H_2O = 3/7).

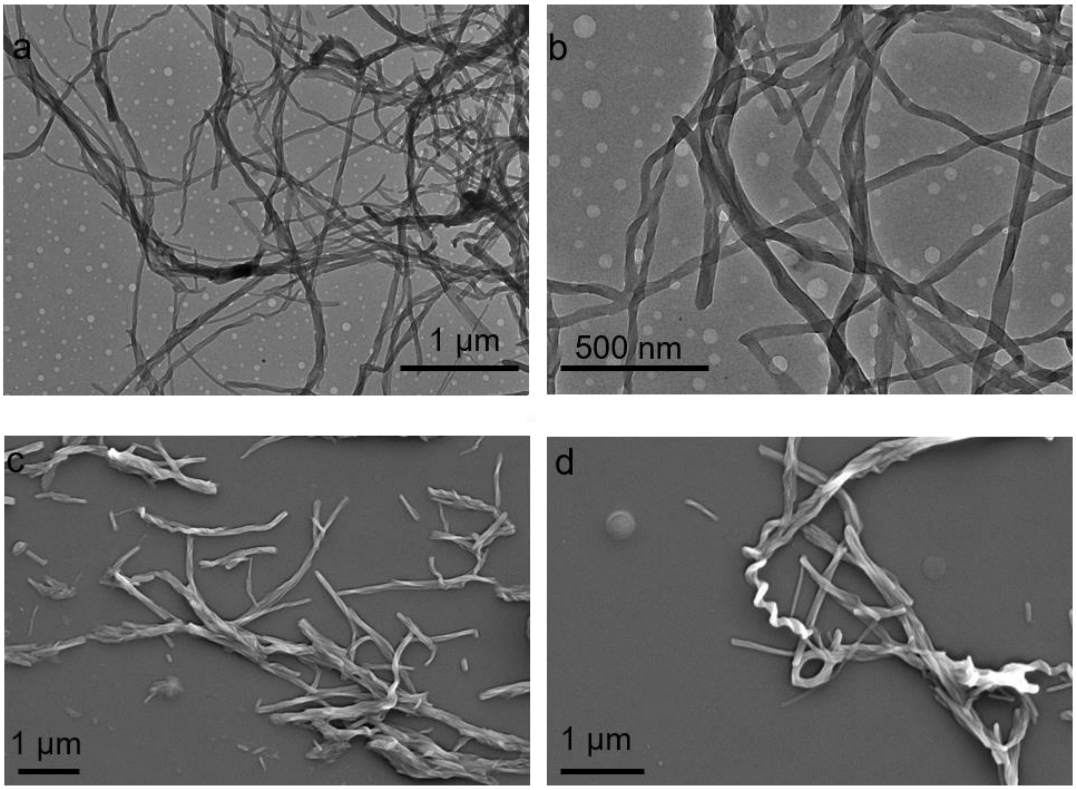

Supplementary Fig. 13. TEM **a, b** and SEM **c, d** images of PNC with 0.3 mol% CC (2×10^{-4} M, THF/H_2O = 3/7).

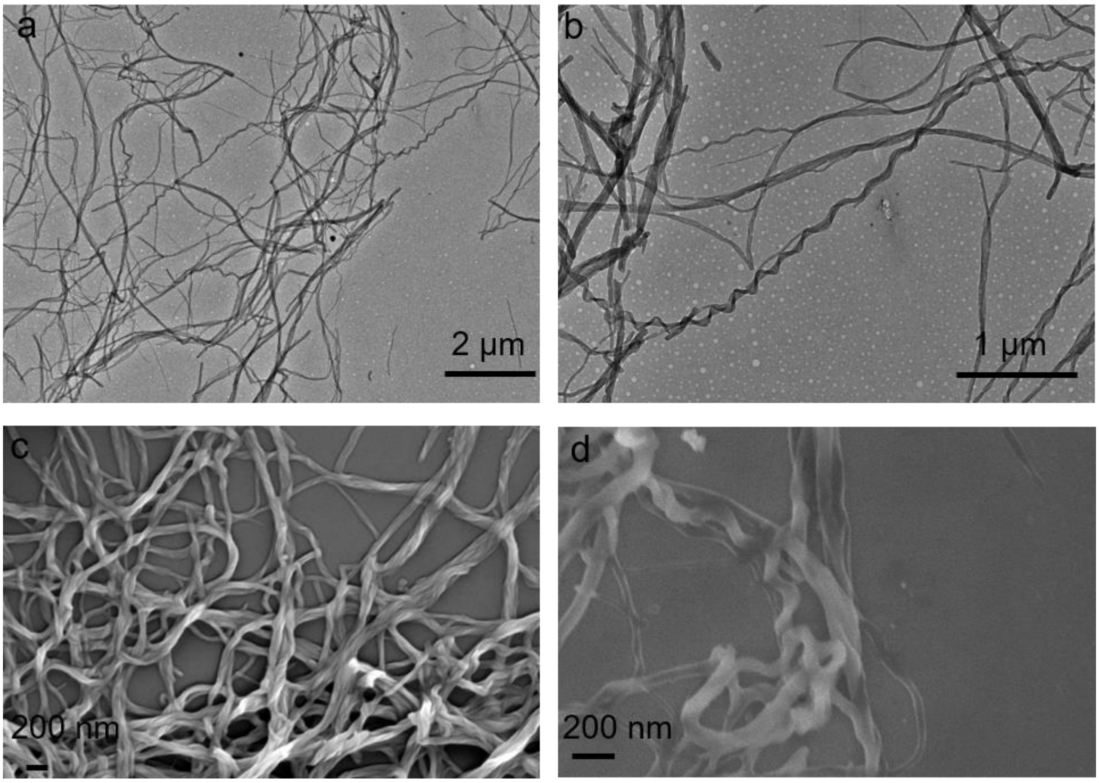

Supplementary Fig. 14. TEM **a, b** and SEM **c, d** images of PNC with 10 mol% CC (2×10^{-4} M, THF/H_2O = 3/7).

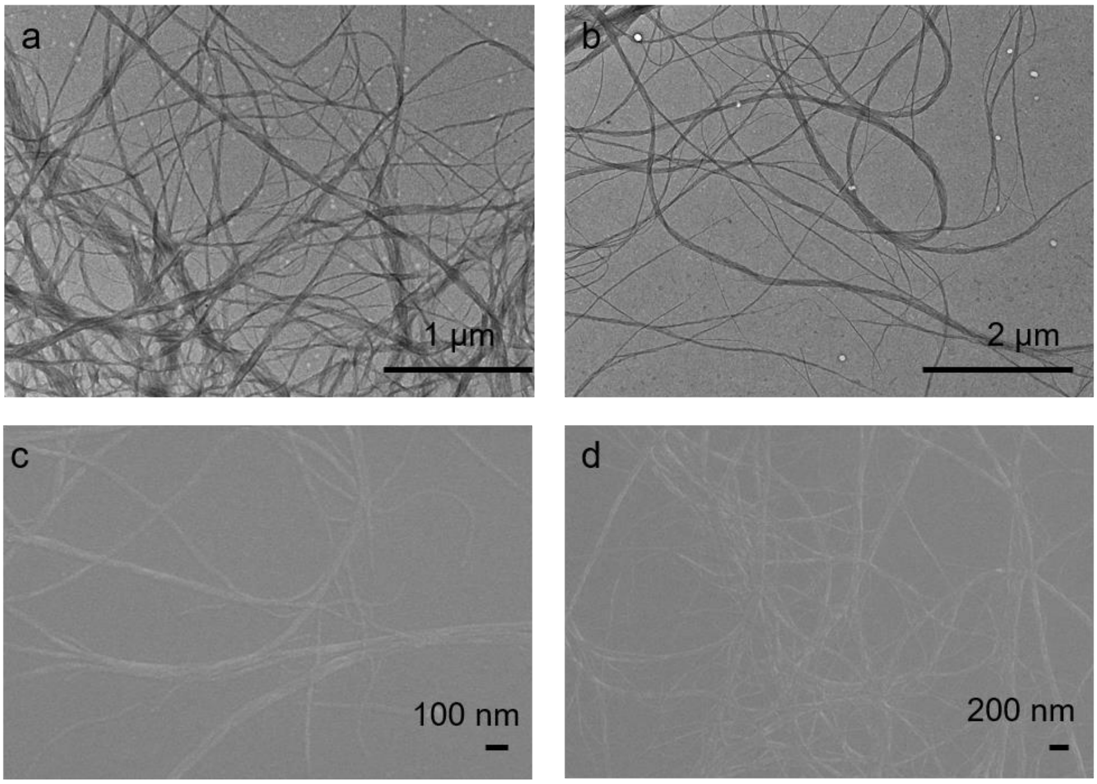

Supplementary Fig. 15. TEM **a, b** and SEM **c, d** images of PNC self-assemblies (10^{-3} M, THF/Decane = 1/9).

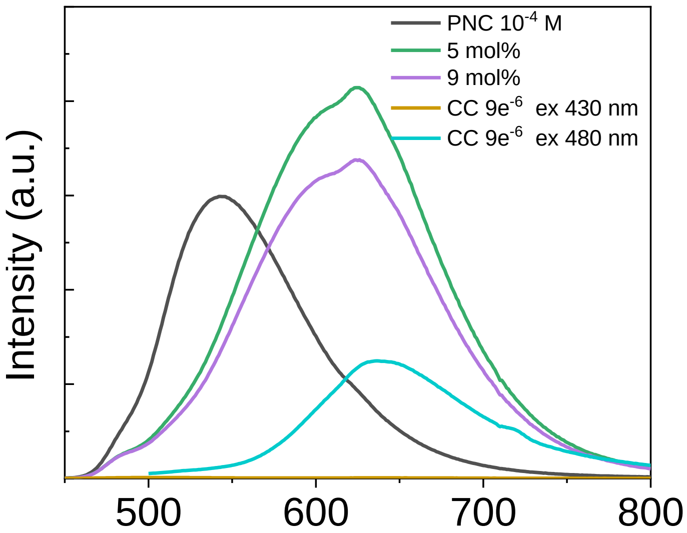

Supplementary Fig. 16. Fluorescent emission spectra of PNC self-assemblies with different CC molar ratios (10^{-4} M, THF/H_2O = 1/9).

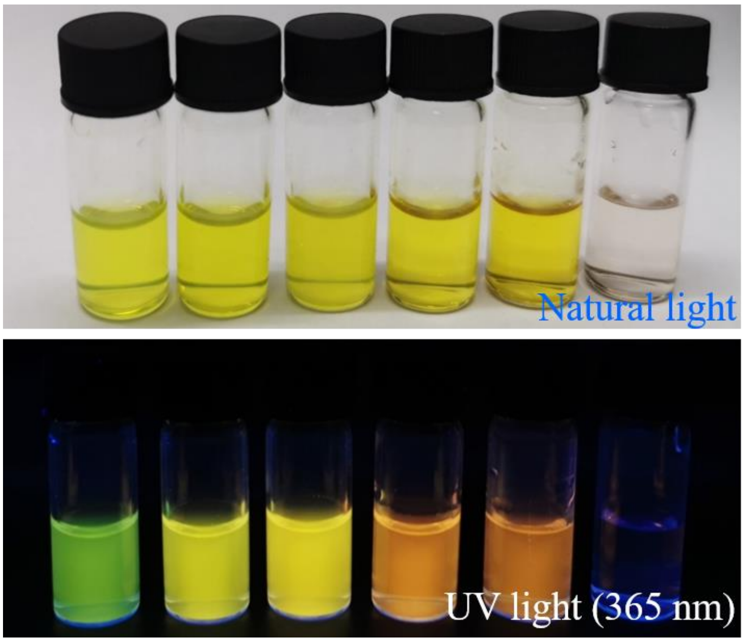

Supplementary Fig. 17. Pictures of PNC (10^{-4} M) self-assemblies with different CC molar ratios (0 mol%, 0.3 mol%, 0.5 mol%, 5 mol%, 9 mol% and pure CC (9x10^{-6} M, THF/H_2O = 1/9).

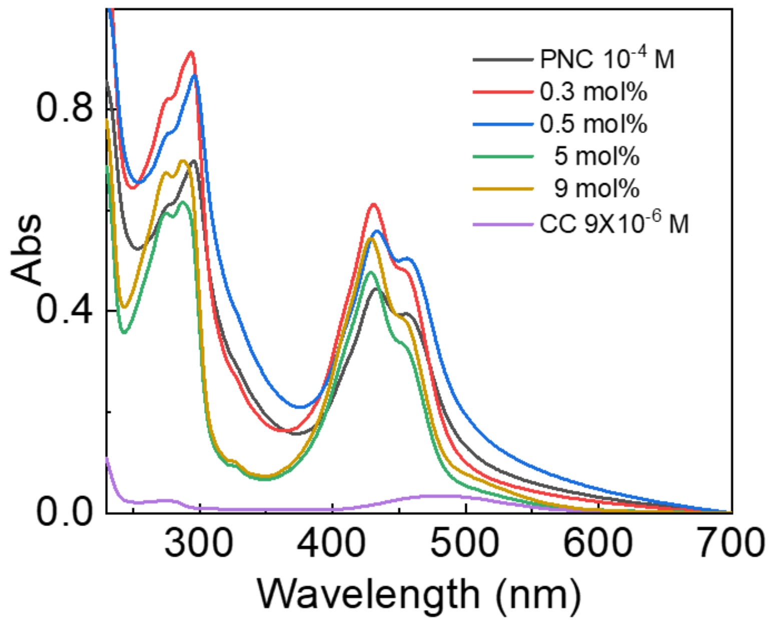

Supplementary Fig. 18. UV-Vis absorption spectra of PNC self-assemblies with different CC molar ratios (10^{-4} M, THF/H_2O = 1/9).

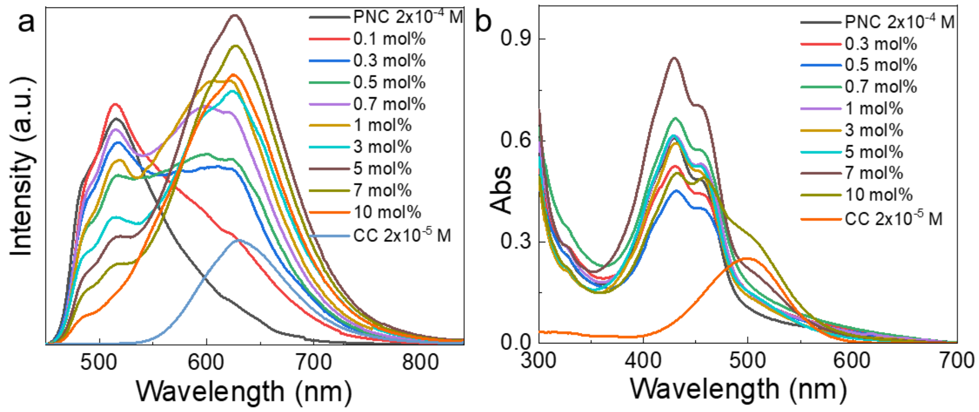

Supplementary Fig. 19. Fluorescent emission and UV-Vis absorption spectra of PNC self-assemblies with different CC molar ratios ([PNC] = 2×10^{-4} M, THF/H_2O = 3/7).

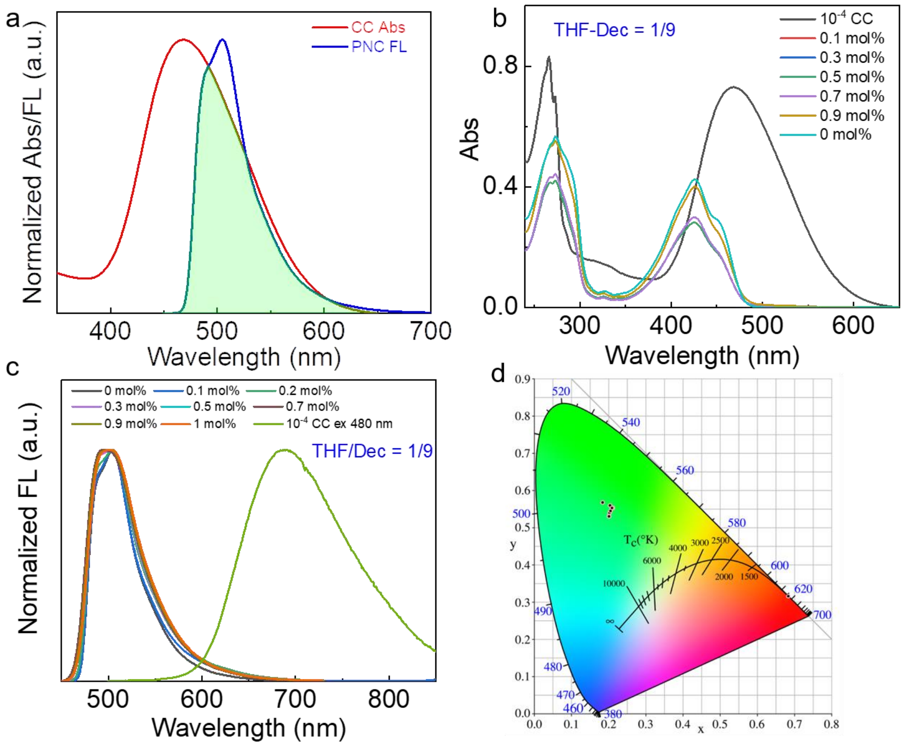

Supplementary Fig. 20. a Spectrum overlaps of donor emission ([PNC] = 10^{-3} M) and acceptor ([CC] = 10^{-4} M) absorption in THF/Decane = 1/9. **b** UV-Vis absorption and **c** Fluorescent emission spectra of PNC (10^{-3} M) self-assemblies with different CC molar ratios and **d** the corresponding CIE coordinate.

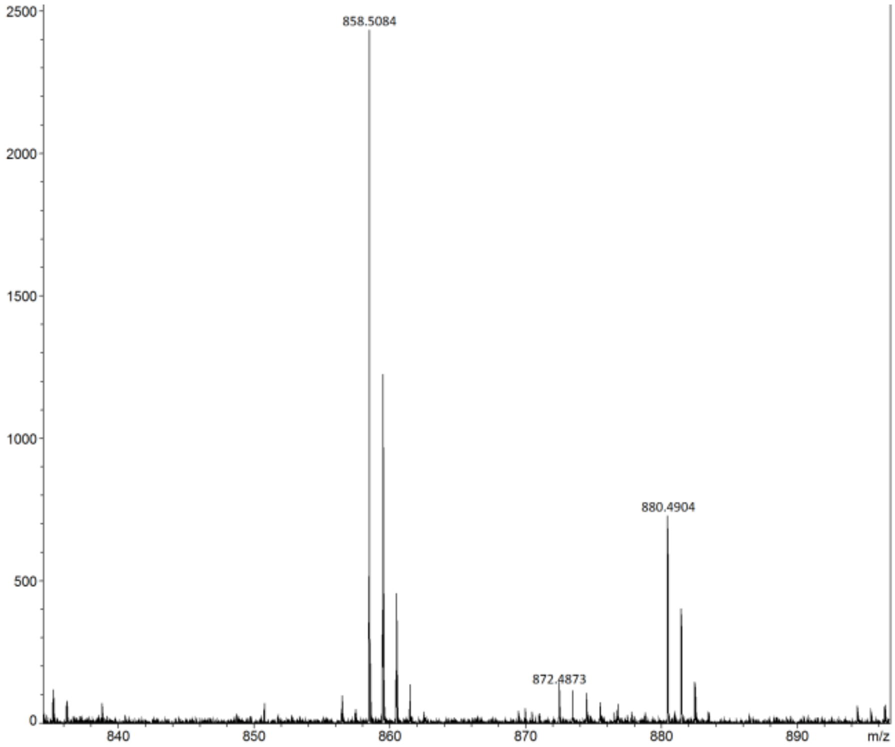

Supplementary Fig. 21. HR-MS spectrum of CC treating with Na_2SO_3.

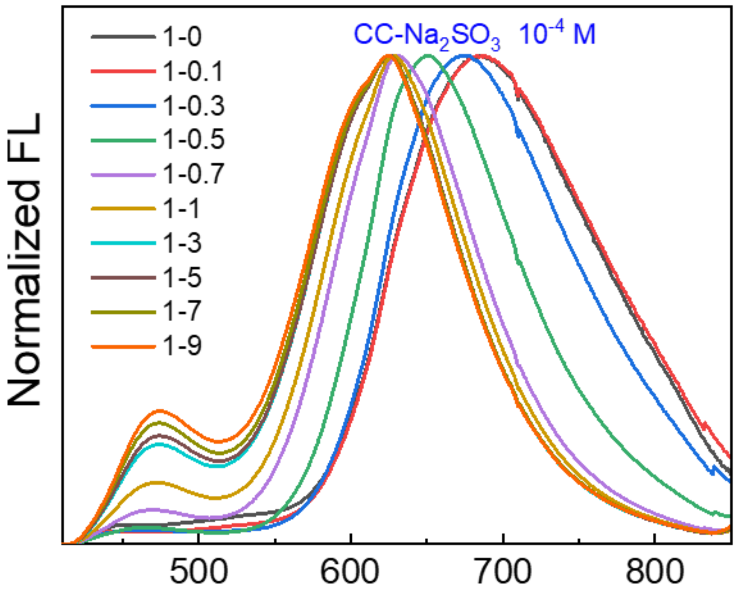

Supplementary Fig. 22. Normalized fluorescent emission of CC assemblies (10^{-4}, THF/H_2O = 1/9) with various concentration of Na_2SO_3 (λ_{ex} = 390 nm).

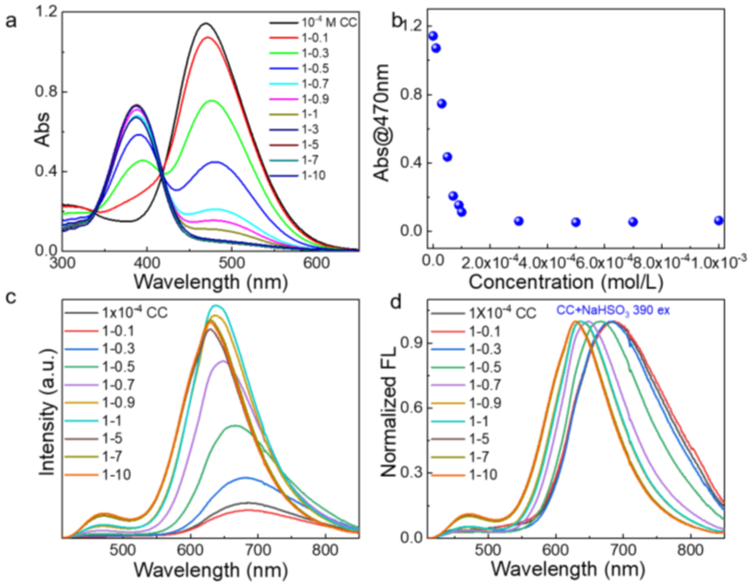

Supplementary Fig. 23. **a** UV-Vis absorption and **b** corresponding vibration of absorption at 470 nm of CC (10^{-4} M) with various concentration of $NaHSO_3$. **c** Fluorescent emission and normalized fluorescent emission of CC with various concentration of $NaHSO_3$ in THF/H_2O = 1/9 (excited at 390 nm).

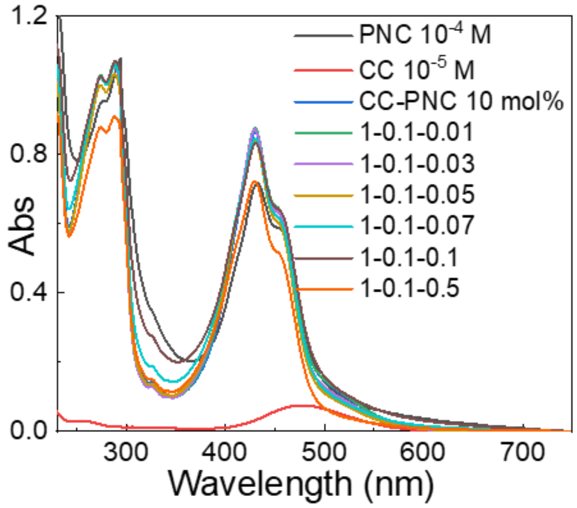

Supplementary Fig. 24. UV-Vis absorption spectra of PNC/CC coassemblies ([PNC] = 10^{-4} M, [CC] = 10^{-5} M) with various concentration of Na_2SO_3 (THF/H_2O = 1/9).

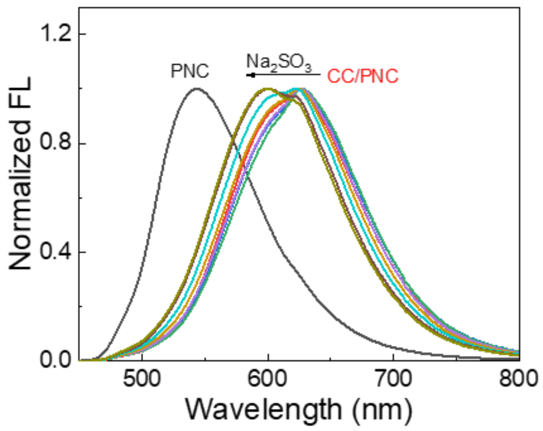

Supplementary Fig. 25. Normalized fluorescent emission spectra of PNC/CC coassemblies ([PNC] = 10^{-4} M, [CC] = 10^{-5} M) with various concentration of Na_2SO_3 (1×10^{-6} M, 3×10^{-6} M, 5×10^{-6} M, 7×10^{-6} M, 1×10^{-5} M, 3×10^{-5} M, 5×10^{-5} M, THF/H_2O = 1/9)

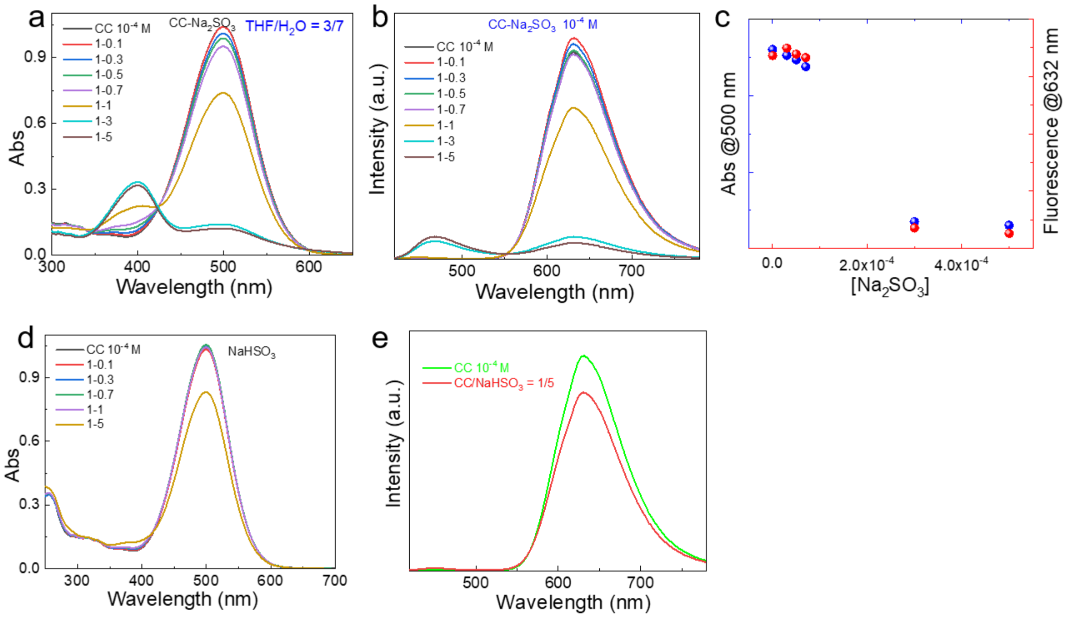

Supplementary Fig. 26. **a** UV-Vis absorption and **b** fluorescent emission spectra of CC assemblies with various concentration of Na_2SO_3. **c** UV-Vis absorption and **d** fluorescent emission spectra of CC assemblies with various concentration of $NaHSO_3$ (THF/H_2O = 3/7, λ_{ex} = 400 nm).

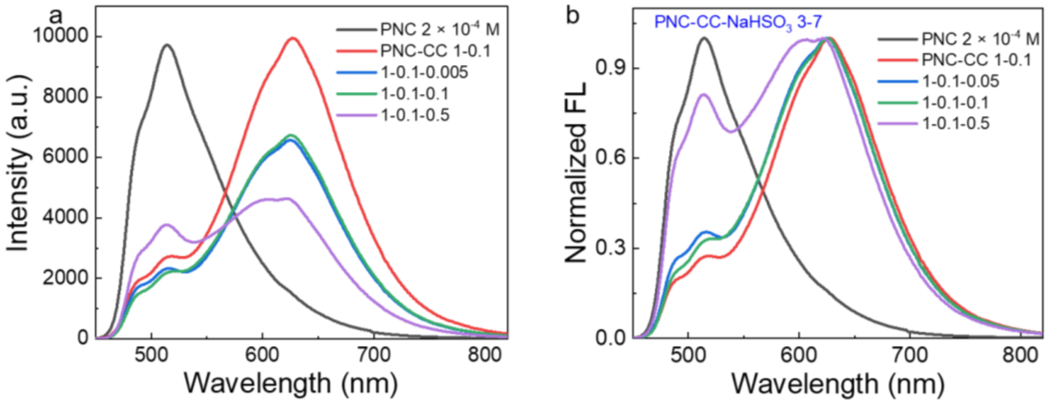

Supplementary Fig. 27. Fluorescent **a** and Normalized fluorescent emission spectra **b** of PNC/CC coassemblies ([PNC] = 2 × 10^{-4}, [CC] = 2 × 10^{-5}]) with various concentration of $NaHSO_3$ (THF/H_2O = 3/7, λ_{ex} = 430 nm).

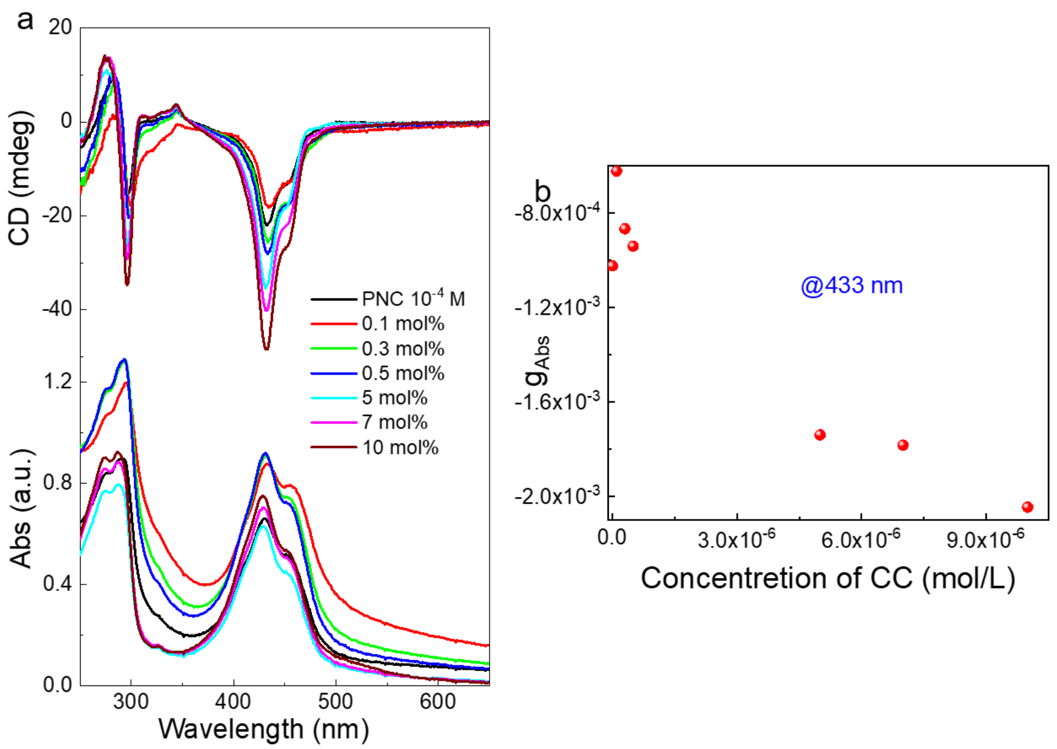

Supplementary Fig. 28. a CD and corresponding UV-Vis absorption spectra of PNC assembies (10^{-4} M) with various amount of CC. **b** Vibration of g_{abs} about PNC/CC coassemblies at 433 nm (THF/H_2O = 1/9).

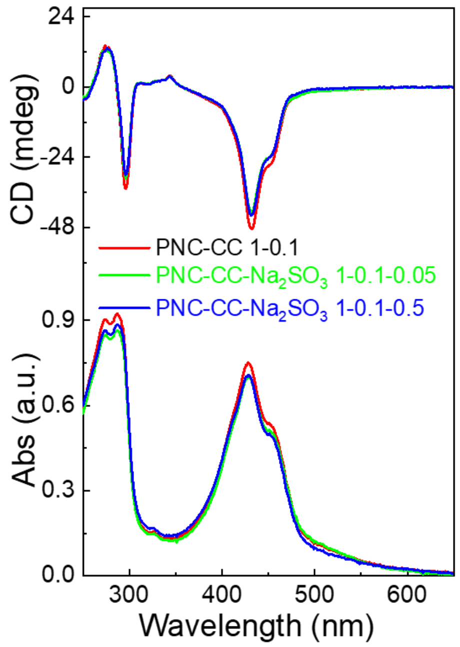

Supplementary Fig. 29. CD spectra of PNC/CC ([PNC] = 10^{-4} M, [CC] = 10^{-5} M) with various concentration of Na_2SO_3 (THF/H_2O = 1/9).

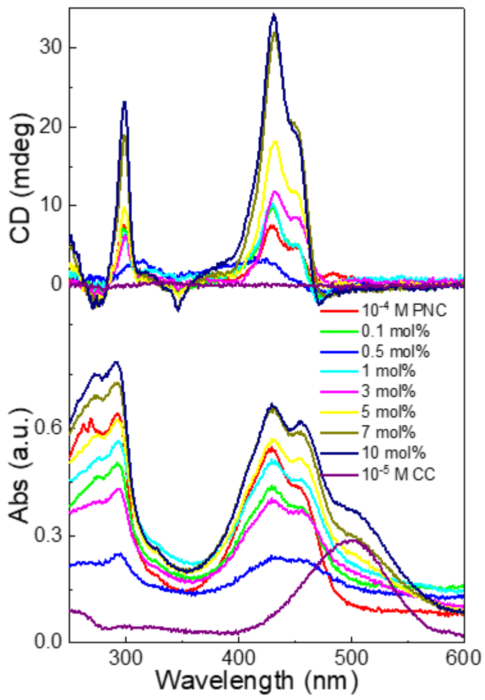

Supplementary Fig. 30. CD and corresponding UV-Vis absorption spectra of PNC assemblies ([PNC] = 10^{-4} M) with various amount of CC (THF/H_2O = 3/7).

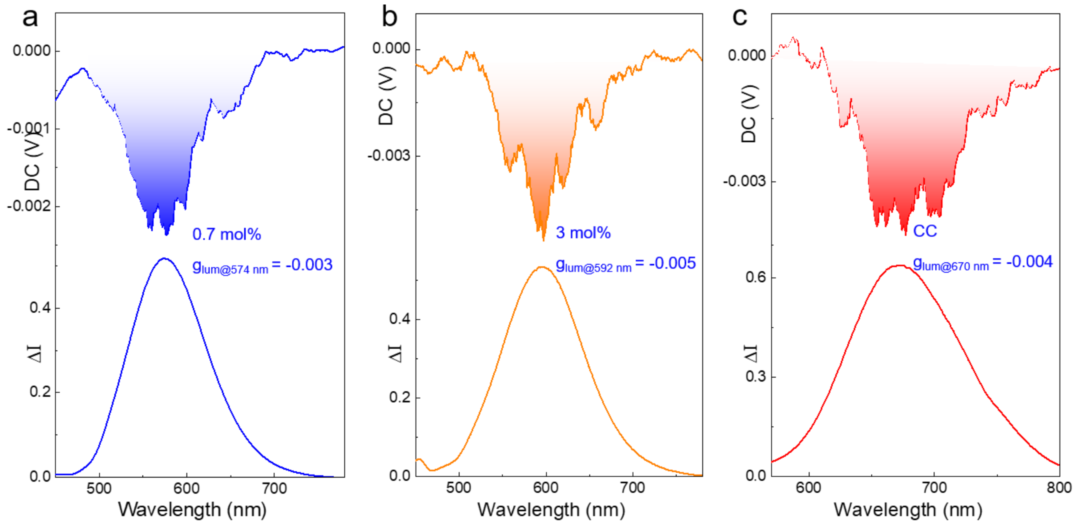

Supplementary Fig. 31. CPL spectra and corresponding fluorescent emission of PNC assemblies with CC. **a** 0.7 mol%, **b** 3 mol% (film). (excited at 430 nm, [PNC] = 10^{-4} M). **c** CPL spectrum of CC self-assemblies (film) (excited at 480 nm) (Film from assemblies forming with THF/H_2O = 1/9, [CC] = 10^{-4} M).

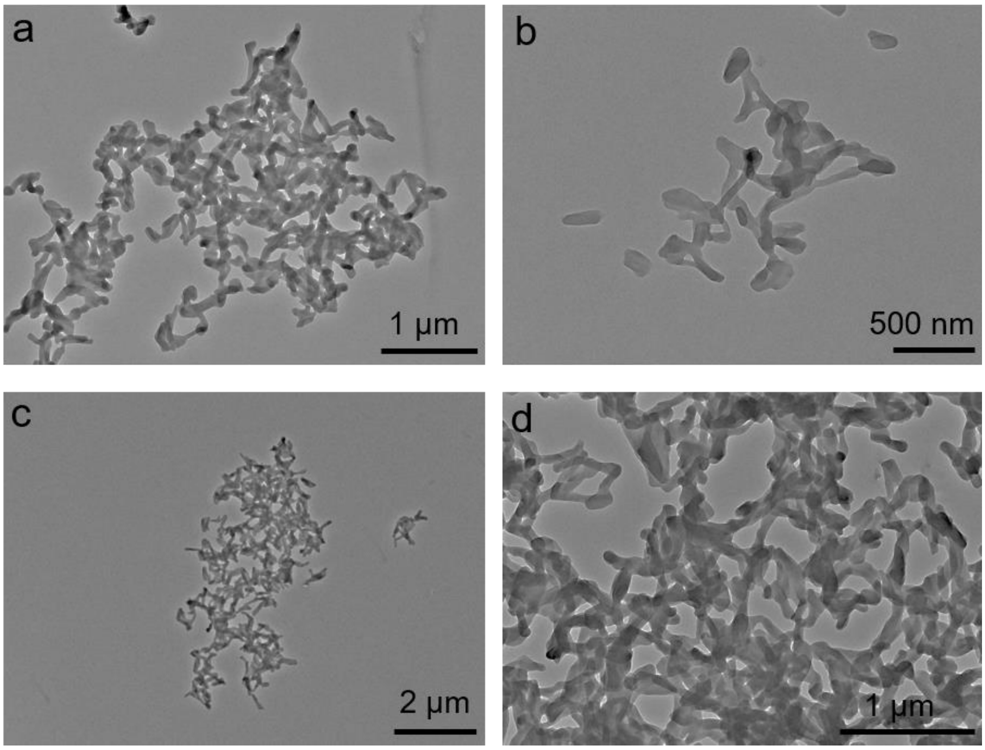

Supplementary Fig. 32 TEM images of **a, b** PNC/CC/Na_2SO_3 = 1/0.1/0.5 and **c, d** PNC/CC/$NaHSO_3$ = 1/0.1/0.5 ([PNC] = 10^{-4} M, THF/H_2O = 1/9).

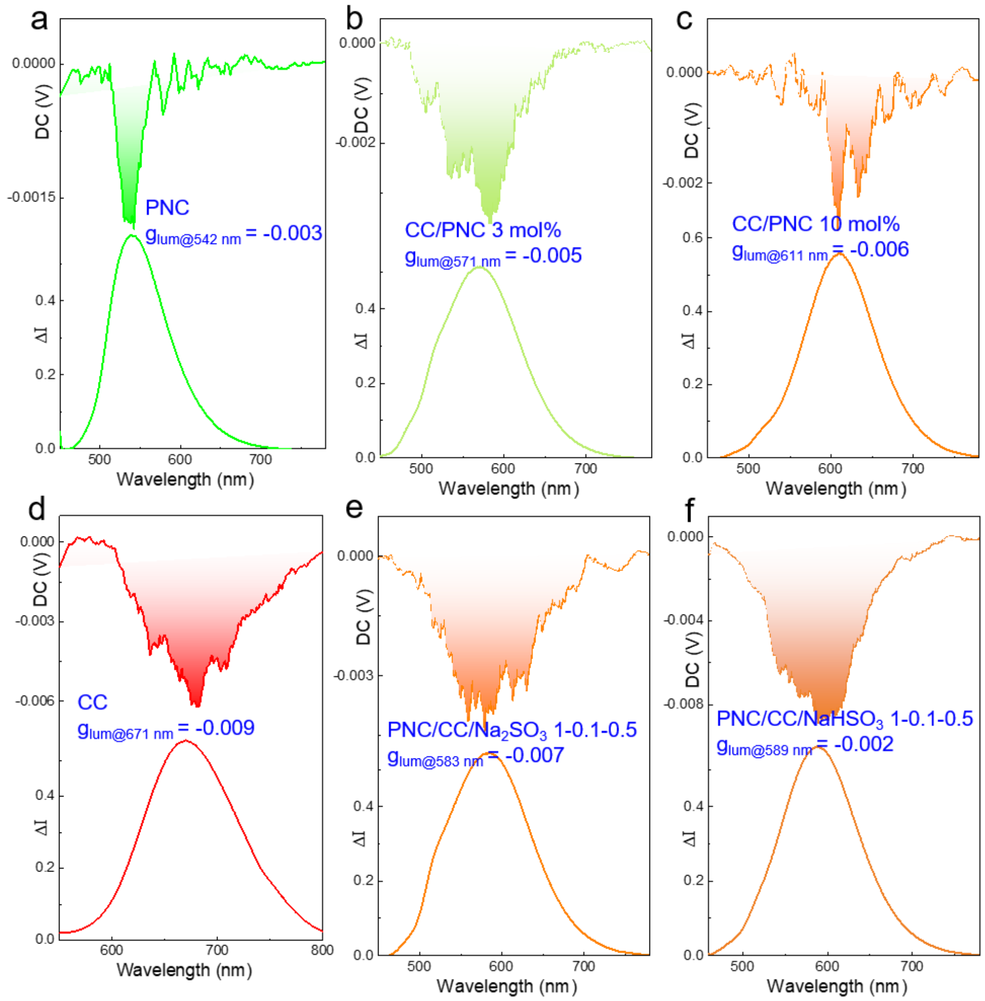

Supplementary Fig. 33. CPL and corresponding fluorescent emission spectra of PNC assemblies with CC. **a** PNC, **b** 3 mol%, **c** 10 mol% (excited at 430 nm), **d** CC (excited at 480 nm), **e** PNC/CC/Na_2SO_3 = 1/0.1/0.5, **f** PNC/CC/$NaHSO_3$ = 1/0.1/0.5 (excited at 430 nm, [PNC] = 10^{-4} M, THF/H_2O = 3/7).

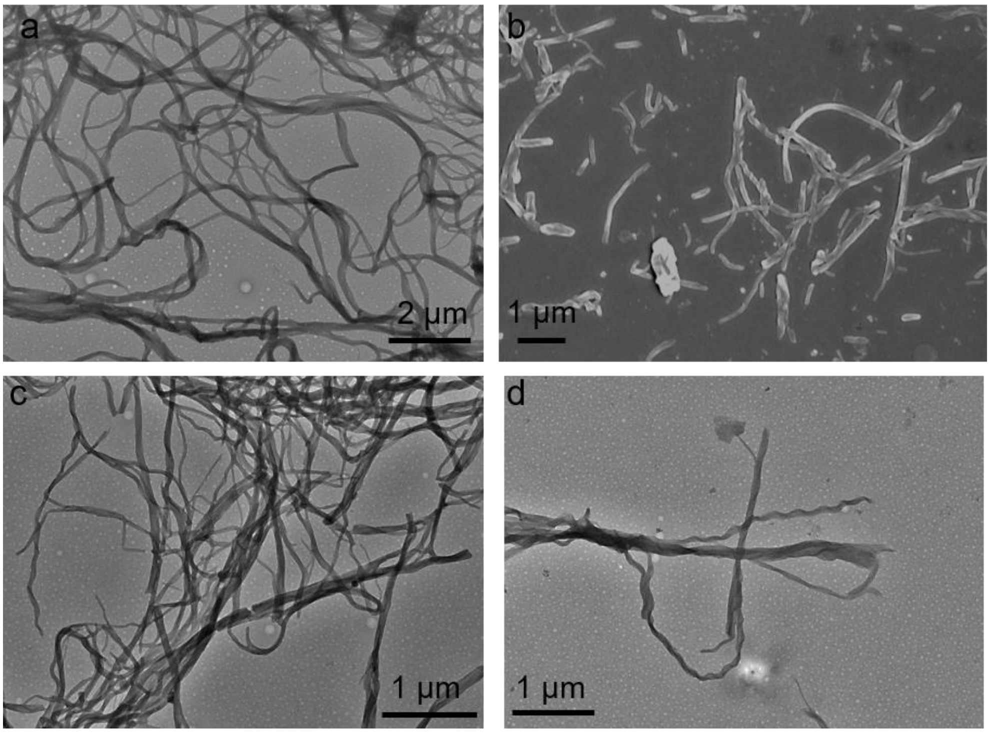

Supplementary Fig. 34. TEM **a** and SEM **b** images of PNC/CC/Na_2SO_3 = 1/0.1/0.5. **c, d** TEM images of PNC/CC/$NaHSO_3$ = 1/0.1/0.5 ([PNC] = 2 × 10^{-4} M, THF/H_2O = 3/7).

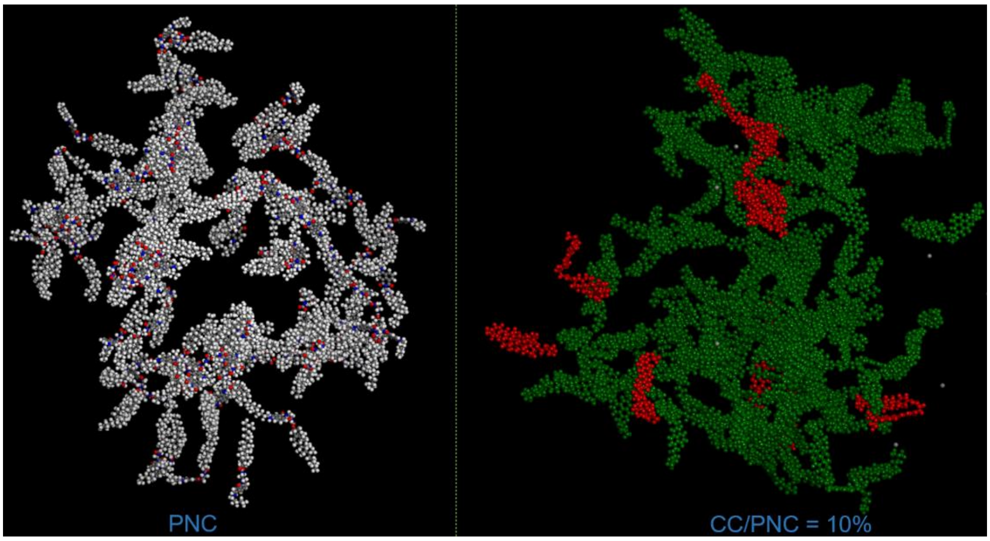

Supplementary Fig. 35. MD simulation results (50 ns) of PNC and PNC/CC coassembly (THF/H_2O = 3/7).

Supplementary References

1. Yuan, H., Wu, Y., Pan, X., Gao, L. & Xiao, G. Pyridyl ionic liquid functionalized zif-90 for catalytic conversion of CO_2 into cyclic carbonates. *Catalysis Let.* **150**, 3561-3571 (2020).

2. Hou, J. -T., Yang, J., Li, K., Liao, Y., Yu, K., Xie, Y.-M. & Yu, X.-Q. A highly selective water-soluble optical probe for endogenous peroxynitrite. *Chem. Commun*. **50**, 9947-9950 (2014).

3. Cheng, Q., Duan, H., Hao, A. & Xing, P. A photo-regulated "breathing" vesicle with inversed supramolecular chirality. *ACS Appl. Mater. Interfaces* **13**, 2091-2099 (2021).

4. Cao, Z., Wang, B., Zhu, F., Hao, A. & Xing, P. Solvent-Processed circularly polarized luminescence in lightharvesting coassemblies. *ACS Appl. Mater. Interfaces* **12**, 34470-34478 (2020).
